# ﻿Exploring ascomycete diversity in Yunnan II: Introducing three novel species in the suborder Massarineae (Dothideomycetes, Pleosporales) from fern and grasses

**DOI:** 10.3897/mycokeys.104.112149

**Published:** 2024-04-16

**Authors:** Rungtiwa Phookamsak, Sinang Hongsanan, Darbhe Jayarama Bhat, Dhanushka N. Wanasinghe, Itthayakorn Promputtha, Nakarin Suwannarach, Jaturong Kumla, Ning Xie, Turki M. Dawoud, Peter E. Mortimer, Jianchu Xu, Saisamorn Lumyong

**Affiliations:** 1 Center of Excellence in Microbial Diversity and Sustainable Utilization, Chiang Mai University, Chiang Mai 50200, Thailand; 2 Department of Economic Plants and Biotechnology, Yunnan Key Laboratory for Wild Plant Resources, Kunming Institute of Botany, Chinese Academy of Sciences, Kunming 650201, Yunnan Province, China; 3 Honghe Center for Mountain Futures, Kunming Institute of Botany, Chinese Academy of Sciences, Honghe, 654400, Yunnan Province, China; 4 Shenzhen Key Laboratory of Microbial Genetic Engineering, College of Life Sciences and Oceanography, Shenzhen University, Shenzhen 518060, China; 5 Department of Botany and Microbiology, College of Science, King Saud University, P.O. Box 2455, Riyadh 11451, Saudi Arabia; 6 Vishnugupta Vishwavidyapeetam, Ashoke, Gokarna 581326, India; 7 CIFOR-ICRAF China Program, World Agroforestry (ICRAF), Kunming 650201, Yunnan Province, China; 8 Center for Mountain Futures (CMF), Kunming Institute of Botany, Chinese Academy of Sciences, Kunming 650201, Yunnan Province, China; 9 Department of Biology, Faculty of Science, Chiang Mai University, Chiang Mai 50200, Thailand; 10 Academy of Science, The Royal Society of Thailand, Bangkok 10300, Thailand

**Keywords:** Ascomycota, *Bambusicola*, *Periconia*, phylogeny, polyphasic approach, taxonomy, the Greater Mekong Subregion, *Trichobotrys*

## Abstract

This article presents the results of an ongoing inventory of Ascomycota in Yunnan, China, carried out as part of the research project series “Exploring ascomycete diversity in Yunnan”. From over 100 samples collected from diverse host substrates, microfungi have been isolated, identified and are currently being documented. The primary objective of this research is to promote the discovery of novel taxa and explore the ascomycete diversity in the region, utilising a morphology-phylogeny approach. This article represents the second series of species descriptions for the project and introduces three undocumented species found in the families Bambusicolaceae, Dictyosporiaceae and Periconiaceae, belonging to the suborder Massarineae (Pleosporales, Dothideomycetes). These novel taxa exhibit typical morphological characteristics of *Bambusicola*, *Periconia* and *Trichobotrys*, leading to their designation as *Bambusicolahongheensis*, *Periconiakunmingensis* and *Trichobotryssinensis*. Comprehensive multigene phylogenetic analyses were conducted to validate the novelty of these species. The results revealed well-defined clades that are clearly distinct from other related species, providing robust support for their placement within their respective families. Notably, this study unveils the phylogenetic affinity of *Trichobotrys* within Dictyosporiaceae for the first time. Additionally, the synanamorphism for the genus *Trichobotrys* is also reported for the first time. Detailed descriptions, illustrations and updated phylogenies of the novel species are provided, and thus presenting a valuable resource for researchers and mycologists interested in the diversity of ascomycetes in Yunnan. By enhancing our understanding of the Ascomycota diversity in this region, this research contributes to the broader field of fungal taxonomy and their phylogenetic understanding.

## ﻿Introduction

Pleosporales is the largest order of Dothideomycetes, comprising two main suborders (viz. Massarineae and Pleosporineae), 91 families, 653 genera (including Pleosporales genera *incertae sedis*) and a quarter of all Dothideomycetes species (Hongsanan et al. 2020; Wijayawardene et al. 2022b). The order was invalidly introduced by Luttrell (1955) and later validated by Barr (1987) and is characterised by perithecial ascomata with typically a papillate ostiole, bitunicate, fissitunicate asci and hyaline to pigmented, variedly shaped, mostly septate ascospores. The asexual morph is represented by both coelomycetes and hyphomycetes (Zhang et al. 2012; Hyde et al. 2013; Hongsanan et al. 2020). Members of Pleosporales are ecologically and morphologically diverse and also shown to be polyphyletic in various groups, as well as contained within species complexes still waiting to be resolved (Zhang et al. 2012; Hyde et al. 2013; Jaklitsch et al. 2016a; Hongsanan et al. 2020). Pleosporalean species are cosmopolitan and ubiquitous in diverse ecological niches. Their life modes include epiphytes, endophytes or parasites on living organisms, hyperparasites on fungi or insects, saprobes, pathogens and lichenised fungi (Zhang et al. 2012; Hyde et al. 2013; Tanaka et al. 2015; Jaklitsch et al. 2016a; Hongsanan et al. 2020). Of these, several genera, such as *Alternaria*, *Bipolaris*, *Didymella*, *Leptospharia*, *Parastagonospora*, *Phaeosphaeria* and *Pyrenophora*, have been reported as plant pathogens causing severe diseases on economic crops (Quaedvlieg et al. 2013; Woudenberg et al. 2013, 2014, 2015; Manamgoda et al. 2014; Ariyawansa et al. 2015a, b; Chen et al. 2015, 2017; Tanaka et al. 2015; El-Demerdash 2018; Khiralla et al. 2019; Bhunjun et al. 2020; Hongsanan et al. 2020; Backes et al. 2021; Bartosiak et al. 2021; Li et al. 2023).

A comprehensive study of the genera in Pleosporales was carried out by Zhang et al. (2012), based on morphological studies of the type specimens coupled with phylogenetic analyses. Consequently, the taxonomic treatment of numerous Pleosporales was updated by various authors, based on polyphasic taxonomic approaches, mainly using morphology-phylogeny-based taxonomy (Ariyawansa et al. 2014, 2015a, b; Phookamsak et al. 2014, 2015; Tanaka et al. 2015; Thambugala et al. 2015; Boonmee et al. 2016; Jaklitsch and Voglmayr 2016; Jaklitsch et al. 2016a, b, 2018; Su et al. 2016; Chen et al. 2017; Hashimoto et al. 2017; Wanasinghe et al. 2017a, b). Even though novel taxa of Pleosporales have been dramatically increasing over the last ten years after the taxonomic circumscription provided by Zhang et al. (2012) and Hyde et al. (2013), there is still over a quarter of the total known species lacking molecular data and/or reliable phylogenetic markers for clarifying the placements in Pleosporales.

Yunnan is known as one part of the 36 global biodiversity hotspots where over 17,000 species of vascular plants are known, including highly endemic species (Feng and Yang 2018; Cai et al. 2019). Highly diverse environments and geographical distribution, as well as flourishing vegetation, have shown the Province to be one of the richest sources of fungi, covering over 40% of the known species in China (Feng and Yang 2018; Liu et al. 2018). Feng and Yang (2018) estimated a species number of fungi existing in Yunnan Province, based on the ratio of local vascular plants and fungi (1:6) following the suggestion of Hawksworth (2001). With this estimation, Yunnan may harbour over 104,000 fungal species; of which only 6000 described species have been reported from the Province, including approximately 3000 species of Ascomycota and Basidiomycota (Feng and Yang 2018).

Since Feng and Yang (2018) updated the status of fungal diversity in this region, the taxonomic study of ascomycetes has steadily increased and over 300 novel species have been discovered in the last five years (Luo et al. 2019; Phookamsak et al. 2019; Dong et al. 2020; Hyde et al. 2020a, b; Wanasinghe et al. 2020, 2022; Wang et al. 2020; Mortimer et al. 2021; Wijayawardene et al. 2021b, 2022a; Gu et al. 2022; Jiang et al. 2022; Yang et al. 2022a, b; Si et al. 2023). However, most studies were restricted to certain groups of ascomycetes, such as bambusicolous fungi (Jiang et al. 2019, 2021b; Dai et al. 2022; Phookamsak et al. 2022), cordycipitoid fungi (Wang et al. 2020; Fan et al. 2021; Dong et al. 2022; Tang et al. 2023), endolichenic fungi (Si et al. 2021, 2023), lignicolous freshwater fungi (Luo et al. 2018a, b, 2019; Su et al. 2018; Dong et al. 2020; Shen et al. 2022), nematode-trapping fungi (Zhang et al. 2020, 2022a, b, c, 2023; Yang et al. 2023b) and woody litter-inhabiting fungi (Mortimer et al. 2021; Wanasinghe et al. 2022), as well as fungi associated with specific host plants (e.g. *Camellia*, *Coffea*, *Magnolia*, *Mangifera* and *Rhododendron*) (Wanasinghe et al. 2020; Gu et al. 2022; Lu et al. 2022; Tibpromma et al. 2022; Wijayawardene et al. 2022a; Yang et al. 2022a, b, 2023a). Comparable with the total estimated number of species that may be found in this region, these fungal inventories are still only representing a small number of extant ascomycetes in Yunnan.

The present study aims to introduce three novel pleosporalean species from Yunnan, based on morphological characteristics and phylogenetic evidence coupled with the differences in nucleotide pairwise comparison amongst closely-related species.

## ﻿Materials and methods

### ﻿Sample collection, isolation, morphological examination and preservation

Samples were collected from Yunnan Province, China during 2016–2021 at three different collecting sites: Honghe (rice terraces), Kunming (botanical garden) and Xishuangbanna (secondary forest). Specimens were collected during the rainy (September) and dry seasons (January and April) and brought to the laboratory in sealed plastic Ziploc bags for further observation and examination. The samples were observed and axenic cultures, via single spore isolation, were obtained within 1–2 weeks after collection. Single spore isolation was performed using the spore suspension technique (Senanayake et al. 2020). Two sets (five spores per set) of the germinated spores were placed separately on to freshly sterilised potato dextrose agar (PDA) medium and incubated under normal day/night light conditions at room temperature (15–25 °C depending on the rainy and dry seasons). Culture characteristics, growth and sporulation *in vitro* were observed and recorded after one and four-week intervals.

Macro-morphological features, such as ascomata and fungal colonies visualised on host substrates, were observed using an Olympus SZ61 series stereomicroscope and photo-captured by a digital camera. Micro-morphological features were examined by differential interference contrast (DIC) microscopy using a Nikon ECLIPSE Ni-U compound microscope and images captured with a Nikon DS-Ri2 camera. The mucilaginous sheath that covered the ascospores was checked by staining with India Ink and the fungal centrum was stained using Congo red for checking the clearity of conidiophores and conidiogenous cells. Lactoglycerol was added to preserve important morphological features on permanent slides. All morphological features were measured using Tarosoft (R) Image FrameWork version 0.9.7. and photographic plates were edited and combined using Adobe Photoshop CS6 software (Adobe Systems Inc., San Jose, CA, USA).

Axenic living cultures were preserved in PDA and sterilised double-distilled water (ddH_2_O) at 4 °C for short-term storage and long-term glycerol storage at -20 °C and -80 °C, respectively. Ex-type living cultures were deposited at the collection of Rungtiwa Phookamsak housed at Honghe Center for Mountain Futures (RPC) and duplicated in the Culture Collection of the Herbarium of Cryptogams Kunming Institute of Botany, Academia Sinica (**KUNCC**), Kunming, China Mae Fah Luang University Culture Collection (**MFLUCC**), Chiang Rai, Thailand. The type specimens were preserved with silica gel and deposited in the Herbarium of Cryptogams Kunming Institute of Botany Academia Sinica (**KUN-HKAS**), China. Index Fungorum numbers (http://www.indexfungorum.org; accessed on 25 May 2023) were obtained for the newly-described taxa.

### ﻿DNA extraction, PCR amplification and sequencing

Fungal genomic DNA was extracted from fresh mycelia using the Biospin Fungus Genomic DNA Extraction Kit (BioFlux, Hangzhou, China) following the procedure from the manufacturer. The genomic DNA was also extracted from ascomata using a Forensic DNA Kit (Omega, Norcross, GA, USA) in case the fungus could not be obtained from the pure culture. Amplicons were generated by polymerase chain reaction (PCR) using five phylogenetic markers, including the internal transcribed spacers region of ribosomal DNA (ITS; ITS1-5.8S-ITS2), the partial 28S large subunit nuclear ribosomal DNA (LSU), the partial 18S small subunit rDNA (SSU), the partial RNA polymerase II second largest subunit (*rpb2*) and the partial translation elongation factor 1-alpha (*tef1-α*). The ITS region was amplified with the primer pair ITS4 and ITS5 (White et al. 1990), the LSU region with LR0R and LR5 (Vilgalys and Hester 1990), the SSU region with NS1 and NS4 (White et al. 1990), the *rpb2* region with fRPB2-5F and fRPB2-7cR (Liu et al. 1999) and the *tef1-α* region with EF1-983F and EF1-2218R (Rehner and Buckley 2005). The component of PCR reaction was performed in a total volume of 25 μl, containing 2 μl DNA template (30–50 ng/μl), 1 μl of each forward and reverse primer (10 μM), 12.5 μl Master Mix (mixture of *EasyTaq*TM DNA Polymerase, dNTPs and optimised buffer; Beijing TransGen Biotech Co., Ltd., Chaoyang District, Beijing, China) and 8.5 µl of double-distilled water (ddH_2_O). The thermal cycle of PCR amplification for ITS, LSU, SSU, *rpb2* and *tef1-α* was set up following Phookamsak et al. (2014, 2023). PCR products were purified and sequenced by using PCR primers at TsingKe Biological Technology (Kunming City, Yunnan Province, China). The quality of raw sequence data was checked and trimmed of low-quality segments with BioEdit 7.1.3.0 (Hall 1999). The consensus sequences of the newly-generated strains were assembled using SeqMan Pro version 11.1.0 (DNASTAR, Inc. Madison, WI, USA) and submitted to the GenBank database to further encourage accession within the scientific community.

### ﻿Sequence alignments and phylogenetic analyses

The newly-generated sequences were subjected to the nucleotide BLAST search tool on the NCBI website for checking the correctness of species identification and searching for closely-related taxa that were further included in the sequence alignment dataset. Reference sequences from relevant publications and BLAST results of the closely-related species were downloaded from GenBank to supplement the datasets (Tables 1–3). Three datasets were prepared to construct the phylogenetic trees for clarifying phylogenetic relationships of the novel taxa in Bambusicolaceae (Table 1), Dictyosporiaceae (Table 2) and Periconiaceae (Table 3). The individual gene dataset was aligned using MAFFT v.7 (Katoh et al. 2019) and improved manually where necessary in Bioedit 7.1.3.0 (Hall 1999). The alignments of individual gene datasets were prior analysed by Maximum Likelihood (ML) for checking the congruence of tree topologies and further combined into a multigene dataset. Phylogenetic analyses were performed, based on ML and Bayesian Inference (BI) analyses.

**Table 1.** Species details and GenBank accession numbers used in phylogenetic analysis of *Bambusicola* species (Bambusicolaceae, Pleosporales). The new sequences are indicated in bold and the ex-type strains are indicated by superscript “T”. Missing sequences are indicated by “–”.

Species name	Strain/specimen no.	GenBank accession numbers				
		ITS	LSU	*rpb2*	SSU	*tef1-α*
*Bambusicolaaquatica* ^T^	MFLUCC 18-1031	MT627729	MN913710	MT878462	MT864293	MT954392
*Bambusicolaautumnalis* ^T^	CGMCC 3.24280	OQ427824	OQ427825	OQ507621	OQ427823	OQ507622
*Bambusicolaautumnalis*	UESTCC 23.0001	OQ609612	OQ550210	OQ556791	OQ550209	OQ556792
*Bambusicolabambusae* ^T^	MFLUCC 11-0614	JX442031	JX442035	KP761718	JX442039	KP761722
*Bambusicoladidymospora* ^T^	MFLUCC 10-0557	KU940116	KU863105	KU940163	KU872110	KU940188
*Bambusicoladimorpha* ^T^	MFLUCC 13-0282	KY026582	KY000661	KY056663	KY038354	–
*Bambusicolaficuum* ^T^	MFLUCC 17-0872	–	MT215580	–	MT215581	MT199326
*Bambusicolafusispora* ^T^	MFLUCC 20-0149	MW076532	MW076531	MW034589	MW076529	–
*Bambusicolaguttulata* ^T^	CGMCC 3.20935	ON332909	ON332927	ON383985	ON332919	ON381177
***Bambusicolahongheensis* ^T^**	**BN06/ KUN-HKAS 129042**	**OR233600**	**OR335804**	**OR540736**	**OR501419**	–
*Bambusicolairregulispora* ^T^	MFLUCC 11-0437	JX442032	JX442036	KP761719	JX442040	KP761723
*Bambusicolaloculata* ^T^	MFLUCC 13-0856	KP761732	KP761729	KP761715	KP761735	KP761724
*Bambusicolamassarinia* ^T^	MFLUCC 11-0389	JX442033	JX442037	KP761716	JX442041	KP761725
*Bambusicolapustulata* ^T^	MFLUCC 15-0190	KU940118	KU863107	KU940165	KU872112	KU940190
*Bambusicolananensis* ^T^	MFLUCC 21-0063	NR_176767	NG_081535	–	–	–
*Bambusicolasichuanensis* ^T^	SICAUCC 16-0002	MK253473	MK253532	MK262830	MK253528	MK262828
*Bambusicolasplendida* ^T^	MFLUCC 11-0439	JX442034	JX442038	KP761717	JX442042	KP761726
*Bambusicolasubthailandica* ^T^	SICAU 16-0005	MK253474	MK253533	MK262831	MK253529	MK262829
*Bambusicolathailandica* ^T^	MFLUCC 11-0147	KU940119	KU863108	KU940166	–	KU940191
*Bambusicolatriseptatispora* ^T^	MFLUCC 11-0166	KU940120	KU863109	KU940167	–	–
*Corylicolaitalica*	MFLU 19-0500	MT554925	MT554926	MT590776	MT554923	–
*Corylicolaitalica* ^T^	MFLUCC 20-0111	MT633085	MT626713	MT635596	MT633084	MT590777
*Leucaenicolaaseptata* ^T^	MFLUCC 17-2423	MK347746	MK347963	MK434891	MK347853	MK360059
*Leucaenicolacamelliae* ^T^	NTUCC 18-093-4	MT112302	MT071278	MT743283	MT071229	MT374091
*Leucaenicolaphraeana* ^T^	MFLUCC 18-0472	MK347785	MK348003	MK434867	MK347892	MK360060
*Occultibambusabambusae* ^T^	MFLUCC 13-0855	KU940123	KU863112	KU940170	KU872116	KU940193
*Occultibambusakunmingensis* ^T^	KUN-HKAS 102151	MT627716	MN913733	MT878453	MT864342	MT954407
*Occultibambusasichuanensis* ^T^	CGMCC 3.20938	ON332913	ON332931	ON383989	–	ON381181
*Palmiascomagregariascomum* ^T^	MFLUCC 11-0175	KP744452	KP744495	KP998466	KP753958	–
*Palmiascomaqujingense* ^T^	KUMCC 19-0201	MT477183	MT477185	MT495782	MT477186	–
*Pseudotetraploabambusicola* ^T^	CGMCC 3.20939	ON332915	ON332933	ON383991	ON332923	ON381183
*Pseudotetraploacurviappendiculata* ^T^	JCM 12852	AB524792	AB524608	–	AB524467	–
*Seriascomabambusae* ^T^	KUMCC 21-0021	MZ329039	MZ329035	MZ325470	MZ329031	MZ325468
*Seriascomadidymosporum* ^T^	MFLUCC 11-0179	KU940127	KU863116	KU940173	KU872119	KU940196
*Seriascomayunnanense* ^T^	MFLU 19-0690	–	MN174695	MN210324	MN174694	MN381858
*Versicolorisporiumtriseptatum* ^T^	JCM 14775	AB365596	AB330081	–	AB524501	–
*Versicolorisporiumtriseptatum*	NMX1222	OL741378	OL741318	–	OL741381	–

**Table 2.** Species details and GenBank accession numbers used in phylogenetic analysis of taxa in Dictyosporiaceae (Pleosporales). The new sequences are indicated in bold and the ex-type strains are indicated by superscript “T”. Missing sequences are indicated by “–”.

Species name	Strain/ specimen no.	GenBank accession numbers			
		ITS	LSU	SSU	*tef1-α*
*Anthosulcatisporasubglobosa* ^T^	MFLUCC 17-2065/ MFLU 17-1473	MT310636	NG_073851	MT226705	MT394649
*Aquadictyosporalignicola* ^T^	MFLUCC 17-1318	MF948621	MF948629	–	MF953164
*Aquaticheirosporalignicola* ^T^	RK-2006a/ HKUCC10304	AY864770	AY736378	AY736377	–
*Cheirosporiumtriseriale* ^T^	HMAS 180703	EU413953	EU413954	–	–
*Chromolaenicolananensis* ^T^	MFLUCC 17-1473	MN325015	NG_070942	MN325009	MN335648
*Darksideaalpha* ^T^	CBS 135650	NR_137619	KP184019	KP184049	KP184166
*Dendryphiellafasciculata* ^T^	MFLUCC 17-1074	NR_154044	NG_059177	–	–
*Dendryphiellavariabilis* ^T^	CBS 584.96	LT963453	LT963454	–	–
*Dictyocheirosporabannica* ^T^	KH 332	LC014543	AB807513	AB797223	AB808489
*Dictyocheirosporarotunda* ^T^	MFLUCC 14-0293b	KU179099	KU179100	–	–
*Dictyosporiumbulbosum*	yone 221	LC014544	AB807511	AB797221	AB808487
*Dictyosporiumelegans* ^T^	NBRC 32502	DQ018087	DQ018100	DQ018079	–
*Didymosphaeriarubi-ulmifolii* ^T^	MFLUCC 14-0023	–	KJ436586	KJ436588	–
*Digitodesmiumbambusicola* ^T^	CBS 110279	DQ018091	DQ018103	–	–
*Falciformisporasenegalensis* ^T^	CBS 196.79	MH861195	NG_057981	NG_062928	KF015687
*Fuscosphaeriahungarica* ^T^	DSE883, CBS 147250	MW209054	MW209059	MW209065	MW238843
*Gregaritheciumcurvisporum* ^T^	HHUF 30134	NR_154049	NG_059394	NG_061002	AB808523
*Gregaritheciumcurvisporum*	MS224	LC482117	–	–	–
	DCR17	MZ047572	–	–	–
*Helicascuselaterascus*	KT 2673/ MAFF 243867	AB809626	AB807533	AB797243	AB808508
*Immotthiabambusae* ^T^	KUN-HKAS 112012AI	MW489455	MW489450	MW489461	MW504646
	KUN-HKAS 112012B	MW489457	MW489452	–	–
*Jalapriyapulchra* ^T^	MFLUCC 15-0348	KU179108	KU179109	KU179110	–
*Jalapriyatoruloides* ^T^	CBS 209.65	DQ018093	DQ018104	DQ018081	–
*Katumotoabambusicola* ^T^	KT1517a	LC014560	AB524595	AB524454	AB539108
*Lentitheciumclioninum* ^T^	KT1149A/ HHUF:28199	NR_154137	NG_059391	NG_064845	AB808515
*Lentitheciumpseudoclioninum* ^T^	HHUF 29055	AB809633	NG_059392	NG_064847	AB808521
*Loculosulcatisporathailandica* ^T^	KUMCC 20-0159	MT376742	MT383964	MT383968	MT380476
*Magnicamarosporiumiriomotense* ^T^	HHUF 30125/ KT 2822	NR_153445	NG_059389	NG_060999	AB808485
*Montagnulacirsii* ^T^	MFLUCC 13-0680	KX274242	KX274249	KX274255	KX284707
*Morosphaeriamuthupetensis* ^T^	NFCCI4219	MF614795	MF614796	MF614797	MF614798
*Murilentitheciumclematidis* ^T^	MFLUCC 14-0562	KM408757	KM408759	KM408761	KM454445
*Neodendryphiellamali* ^T^	CBS 139.95	LT906655	LT906657	EF204511	–
*Neodendryphiellamichoacanensis* ^T^	FMR 16098	NR_160583	LT906658	–	–
*Neohelicascusaquaticus*	MFLUCC 10-0918/ KT 1544	AB809627	AB807532	AB797242	AB808507
*Paradictyocheirosporatectonae* ^T^	NFCCI 4878/ AMH 10301	MW854646	MW854647	–	MW854832
*Phaeosphaeriaoryzae* ^T^	CBS 110110	KF251186	KF251689	GQ387530	–
*Phaeosphaeriopsisglaucopunctata* ^T^	MFLUCC 13-0265	KJ522473	KJ522477	KJ522481	MG520918
*Pseudocoleophomabauhiniae* ^T^	MFLUCC 17–2586	MK347736	MK347953	MK347844	MK360076
*Pseudocoleophomacalamagrostidis* ^T^	KT 3284/ HHUF 30450	LC014592	LC014609	LC014604	LC014614
*Pseudoconiothyriumbroussonetiae* ^T^	CBS:145036/ CPC:33570	NR_163377	NG_066331	–	MK442709
*Pseudoconiothyriumtyphicola* ^T^	MFLUCC 16-0123	KX576655	KX576656	–	–
*Pseudocyclothyriellaclematidis* ^T^	MFLUCC 17-2177A	MT310595	MT214548	–	MT394730
*Pseudocyclothyriellaclematidis*	MFLU 16-0280	MT310596	MT214549	–	–
*Pseudodictyosporiumelegans*^T^ (=*Cheiromoniliophoraelegans*)	CBS 688.93	DQ018099	DQ018106	DQ018084	–
*Pseudodictyosporiumthailandica* ^T^	MFLUCC 16-0029	NR_154347	NG_059688	NG_063611	KX259526
*Sajamaeamycophila* ^T^	APA-2999	MK795715	MK795718	–	–
*Sulcatisporaacerina* ^T^	KT 2982	LC014597	LC014610	LC014605	LC014615
*Tingoldiagograminicola* ^T^	KH68	LC014598	AB521743	AB521726	AB808561
*Trichobotryseffusus*	1179	KJ630313	–	–	–
	HNNUZCJ-94	OM281094	–	–	–
	FS524	MN545626	–	–	–
	SYSU-MS4729	MH050972	–	–	–
	DFFSCS021	JX156367	–	–	–
***Trichobotryssinensis* ^T^**	**RPC 21-007/ KUNCC 23-14554**	**OR233595**	**OR335805**	**OR501420**	**OR547995**
*Trichobotrys* sp. [as *Gregarithecium* sp.]	MFLUCC 13-0853	KX364281	KX364282	KX364283	–
	GMB1217	–	–	OM836759	–
*Trematosphaeriapertusa* ^T^	CBS 122368	NR_132040	NG_057809	FJ201991	KF015701
*Verrucoccumcoppinsii* ^T^	E 00814291	MT918785	MT918770	NG_081399	–
*Verrucoccumspribillei* ^T^	SPO 1154	MT918781	MT918764	MT918772	–
*Vikalpaaustraliense*	HKUCC 8797	DQ018092	–	–	–

**Table 3.** Species details and GenBank accession numbers used in phylogenetic analysis of *Periconia* species (Periconiaceae, Pleosporales). The new sequences are indicated in bold and the ex-type strains are indicated by superscript “T”. Missing sequences are indicated by “–”.

Species	Strain No.	GenBank accession numbers			
		ITS	LSU	SSU	*tef1-α*
*Flavomycesfulophazae*	CBS 135664	KP184000	KP184039	KP184081	–
*Flavomycesfulophazae* ^T^	CBS 135761	NR_137960	NG_058131	NG_061191	–
*Lentitheciumaquaticum* ^T^	CBS 123099	NR_160229	NG_064211	NG_016507	GU349068
*Lentitheciumclioninum* ^T^	KT 1149A	LC014566	AB807540	AB797250	AB808515
*Lentitheciumclioninum*	KT 1220	LC014567	AB807541	AB797251	AB808516
*Massarinacisti* ^T^	CBS 266.62	–	AB807539	AB797249	AB808514
*Massarinaeburnea*	CBS 473.64	–	GU301840	GU296170	GU349040
*Morosphaeriaramunculicola*	KH 220	–	AB807554	AB797264	AB808530
*Morosphaeriavelatispora*	KH 221	LC014572	AB807556	AB797266	AB808532
*Periconiaalgeriana* ^T^	CBS 321.79	MH861212	MH872979	–	–
*Periconiaalishanica* ^T^	MFLUCC 19-0145	MW063165	MW063229	–	MW183790
*Periconiaaquatica* ^T^	MFLUCC 16-0912	KY794701	KY794705	–	KY814760
*Periconiaartemisiae* ^T^	KUMCC 20-0265	MW448657	MW448571	MW448658	MW460898
*Periconiaartemisiae*	G1782	MK247789	–	–	–
*Periconiaatropurpurea*	CBS 381.55	MH857524	MH869061	–	–
*Periconiabanksiae* ^T^	CBS 129526	JF951147	NG_064279	–	–
*Periconiabyssoides*	KUMCC 20-0264	MW444854	MW444855	MW444856	MW460895
	MAFF 243869	LC014582	AB807569	AB797279	AB808545
	MFLUCC 17-2292	MK347751	MK347968	MK347858	MK360069
	MFLUCC 18-1553	MK347806	MK348025	MK347914	MK360068
	MFLUCC 20-0172	MW063162	MW063226	–	–
	NCYUCC 19-0314	MW063163	MW063227	–	–
*Periconiacaespitosa* ^T^	LAMIC 110 16	MH051906	MH051907	–	–
*Periconiachengduensis* ^T^	CGMCC 3.23930	OP955987	OP956012	OP956056	OP961453
*Periconiachengduensis*	UESTCC 22.0140	OP955977	OP956002	OP956046	OP961443
*Periconiachimonanthi* ^T^	KUMCC 20-0266	MW448660	MW448572	MW448656	MW460897
*Periconiacircinata*	CBS 263.37	MW810265	MH867413	–	MW735660
*Periconiacitlaltepetlensis* ^T^	ENCB 140251 = IOM 325319.1	MH890645	MT625978	–	–
*Periconiacitlaltepetlensis*	IOM 325319.2	MT649221	MT649216	–	–
*Periconiacookei*	MFLUCC 17-1399	MG333490	MG333493	–	MG438279
	MFLUCC 17-1679	–	MG333492	–	MG438278
	UESTCC 22.013	OP955968	OP955993	OP956037	–
*Periconiacortaderiae* ^T^	MFLUCC 15-0457	KX965732	KX954401	KX986345	KY310703
*Periconiacynodontis* ^T^	CGMCC 3.23927	OP909925	OP909921	OP909920	OP961434
*Periconiacyperacearum* ^T^	CPC 32138	NR_160357	NG_064549	–	–
*Periconiadelonicis* ^T^	MFLUCC 17-2584	–	NG_068611	NG_065770	MK360071
*Periconiadidymosporum* ^T^	MFLU 15-0058	KP761734	KP761731	KP761738	KP761728
*Periconiadigitata*	CBS 510.77	LC014584	AB807561	AB797271	AB808537
*Periconiaelaeidis* ^T^	MFLUCC 17-0087	MG742713	MH108552	MH108551	–
*Periconiaepilithographicola*	MFLUCC 21–0153	OL753687	OL606155	OL606144	OL912948
*Periconiaepilithographicola* ^T^	CBS 144017	NR_157477	–	–	–
*Periconiafestucae* ^T^	CGMCC 3.23929	OP955973	OP955998	OP956042	OP961439
*Periconiagenistae* ^T^	CBS 322.79	MH861213	MH872980	–	–
*Periconiahomothallica* ^T^	CBS 139698/ KT916	AB809645	AB807565	AB797275	AB808541
*Periconiaigniaria*	CBS 379.86	LC014585	AB807566	AB797276	AB808542
*Periconiaimperatae* ^T^	CGMCC 3.23931	OP955984	OP956009	OP956053	OP961450
*Periconiaimperatae*	UESTCC 22.0145	OP955979	OP956004	OP956048	OP961445
***Periconiakunmingensis* ^T^**	**KUMCC 18-0173/ RPC 15-017**	**MH892346**	**MH892399**	**OR225814**	**MH908963**
*Periconialateralis*	CBS 292.36	MH855804	MH867311	–	–
*Periconiamacrospinosa*	CBS 135663	KP183999	KP184038	KP184080	–
	REF144	JN859364	JN859484	–	–
*Periconiaminutissima*	MFLUCC 15-0245	KY794703	KY794707	–	–
	MUT 2887	MG813227	–	–	–
*Periconianeobrittanica* ^T^	CPC 37903	NR_166344	NG_068342	–	–
*Periconiapalmicola* ^T^	MFLUCC 14-0400	–	NG_068917	MN648319	MN821070
*Periconiapenniseti* ^T^	CGMCC 3.23928	OP955971	OP955996	OP956040	OP961437
*Periconiaprolifica* ^T^	CBS 209.64	MH858422	MH870050	–	–
*Periconiapseudobyssoides*	KUMCC 20-0263	MW444851	MW444852	MW444853	MW460894
*Periconiapseudodigitata*	KT 644	MW444852	AB807562	AB797272	AB808538
*Periconiapseudodigitata* ^T^	KT 1395	MW444853	NG_059396	NG_064850	AB808540
*Periconiasahariana* ^T^	CBS 320.79	MW444854	MH872978	–	–
*Periconiasalina* ^T^	GJ374/ MFLU 19–1235	MW444855	MN017846	MN017912	–
*Periconiaspodiopogonis* ^T^	CGMCC 3.23932	MW444856	OP955988	OP956032	OP961429
*Periconiasubmersa* ^T^	MFLUCC 16-1098	MW444857	KY794706	–	KY814761
*Periconiathailandica* ^T^	MFLUCC 17-0065	MW444858	KY753888	KY753889	–
*Periconiathysanolaenae* ^T^	KUMCC 20-0262	MW444859	MW444850	MW448659	MW460896
*Periconiavariicolor* ^T^	SACCR-64	MW444860	–	–	–
*Periconiaverrucosa* ^T^	MFLUCC 17-2158	MT310617	MT214572	MT226686	MT394631
*Periconiaverrucosa*	UESTCC 22.0136	OP955966	OP955991	OP956035	OP961432
	KT 1825	–	AB807573	AB797283	AB808549
	KT 1820A	–	AB807572	AB797282	AB808548

Maximum Likelihood (ML) implemented by the Randomised Axelerated Maximum Likelihood (RAxML), was performed in RAxML-HPC v.8 on the XSEDE (8.2.12) tool via the online web portal CIPRES Science Gateway v. 3.3 (Miller et al. 2010) using default settings, but adjusted with 1000 bootstrap replicates and a gamma-distributed rate variation of a general time reversible model (GTR) was applied. The BI analyses were conducted by MrBayes on XSEDE v. 3.2.7a via the same web portal as in ML, with two parallel runs. The best-fit model of nucleotide substitution was determined by MrModelTest v. 2.3 (Nylander et al. 2008). Six simultaneous Markov chains were run for 1–5 million generations, but stopped automatically when the critical value for the topological convergence diagnostic reached 0.01. Trees were sampled every 100^th^ generation. The initial 10% of sample trees were treated as burn-in (estimated by Tracer v. 1.7; Rambaut et al. (2018)) and discarded. The remaining trees were used to calculate the posterior probabilities in the majority rule consensus tree. The phylograms were visualised using Figtree v. 1.4.0 (Rambaut and Drummond 2012) and backbone trees were laid out and edited in Adobe Illustrator version 20.0.0. software (Adobe Systems Inc., San Jose, CA, USA).

## ﻿Results

### ﻿Phylogenetic analyses

In this study, three phylogenetic analyses were conducted to clarify the phylogenetic placements of our new taxa within the Bambusicolaceae (Analysis 1), Dictyosporiaceae (Analysis 2) and Periconiaceae (Analysis 3), as follows:

#### ﻿Analysis 1

**Figure 1.**
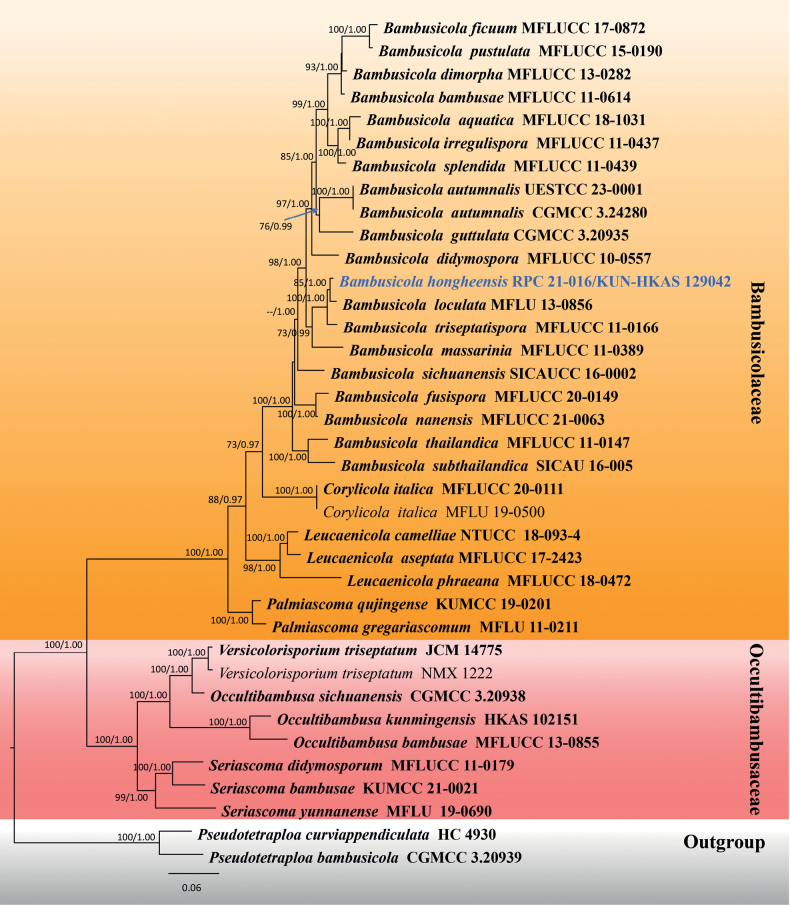
Phylogram of the best-scoring ML consensus tree of taxa in Bambusicolaceae and Occultibambusaceae. The new isolate is indicated in blue. Isolates from type materials are in bold. The ML ultrafast bootstrap and Bayesian PP values greater than 60% and 0.90 are shown at the nodes.

The *Bambusicola* species tree was constructed using a sequence dataset of the concatenated ITS, LSU, *rpb2*, SSU and *tef1-α* of all *Bambusicola* species, as well as representatives of other related genera. A total of 37 strains were included, with two strains of *Pseudotetraploabambusicola* (CGMCC 3.20939) and *P.curviappendiculata* (JCM 12852) as the outgroup. Primarily, phylogenetic analysis of the concatenated LSU, SSU and ITS sequence dataset was conducted, based on ML and compared with the multigene phylogenetic analysis (the concatenated ITS, LSU, *rpb2*, SSU and *tef1-α* sequence dataset). Phylogenetic analysis, based on the concatenated LSU, SSU and ITS gene regions, showed a similar topology with the concatenated ITS, LSU, *rpb2*, SSU and *tef1-α* gene regions and were not significantly different (data not shown). Hence, multigene phylogenetic analysis of the concatenated ITS, LSU, *rpb2*, SSU and *tef1-α* gene regions was selected to represent the phylogenetic relationships of the new species with other closely-related species in Bambusicolaceae. The aligned dataset contained 4929 characters, including gaps. Phylogenetic relationships were inferred by conducting analyses using both ML and BI methods. The best-scoring RAxML tree was selected to represent the relationships amongst taxa, with a final likelihood value of -29592.797597 (Fig. 1). The matrix contained 1905 distinct alignment patterns, with a 22.83% proportion of gaps and completely undetermined characters. The estimated base frequencies of A = 0.243583, C = 0.258293, G = 0.271748, T = 0.226375; substitution rates AC = 1.393909, AG = 2.806593, AT = 1.064133, CG = 1.193703, CT = 6.412290, GT = 1.000000; gamma distribution shape parameter α = 0.589535; Tree-Length = 1.823129. For BI analysis, GTR + I + G was selected as the best-fit model by AIC in MrModelTest for each gene (ITS, LSU, *rpb2*, SSU and *tef1-α*). Six simultaneous Markov chains were set to run for 1,000,000 generations, but stopped at 25,000 generations because the convergence diagnostic hit the stop value, resulting in 251 total trees. The first 10% of trees were discarded as the burn-in phase of the analyses and the remaining trees were used for calculating posterior probabilities in the majority rule consensus tree, of which the final average standard deviation of split frequencies at the end of total MCMC generations was 0.005298.

Multigene phylogenetic analyses demonstrated that all genera of Bambusicolaceae formed well-resolved clades (up to 98% ML, 1.00 PP; Fig. 1) in the present study. The new species, *Bambusicolahongheensis* (KUN-HKAS 129042), clustered with the clade containing *B.loculata* (MFLUCC 13-0856) (85% ML, 1.00 PP) and *B.triseptatispora* (MFLUCC 11-0166) with high statistical support (100% ML, 1.00 PP). These three species have close relationships with *B.massarinia* (MFLUCC 11-0389) (73% ML, 0.99 PP), the type genus of *Bambusicola*.

#### ﻿Analysis 2

**Figure 2.**
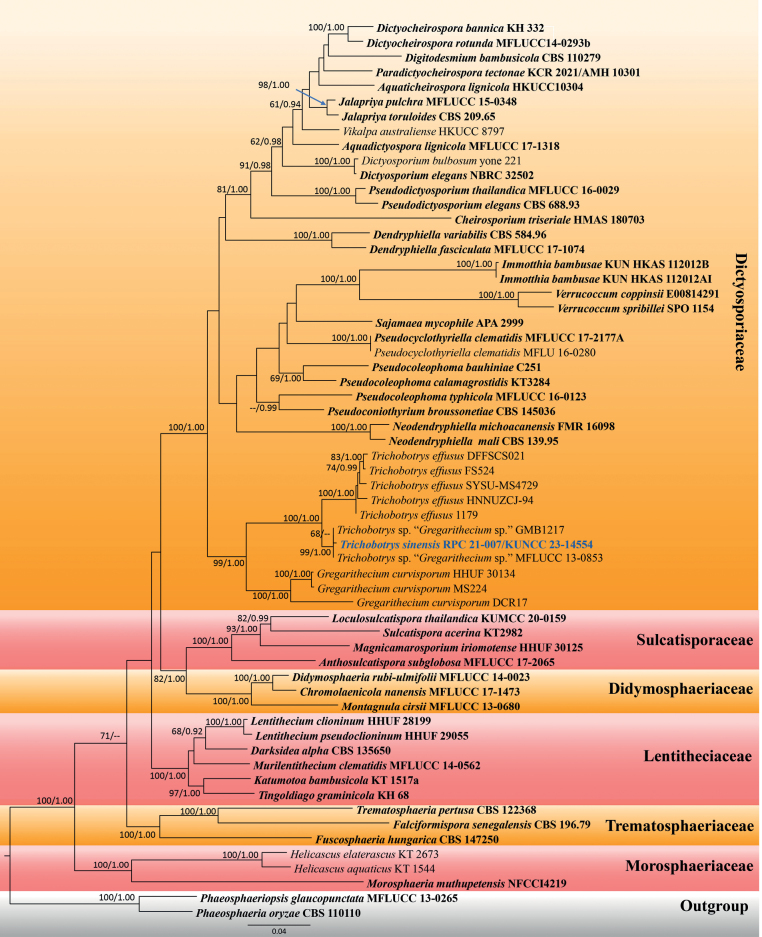
Phylogram of the best-scoring ML consensus tree of *Trichobotrys* species in Dictyosporiaceae and closely-related families viz. Didymosphaeriaceae, Lentitheciaceae, Morosphaeriaceae, Sulcatisporaceae and Trematosphaeriaceae. The new isolate is indicated in blue. Isolates from type materials are in bold. The ML ultrafast bootstrap and Bayesian PP values greater than 70% and 0.95 are shown at the nodes.

The *Trichobotrys* tree was constructed using sequence data from ITS, LSU, SSU and *tef1-α*. A total of 61 strains of taxa in Dictyosporiaceae and closely-related families (viz. Didymosphaeriaceae, Lentitheciaceae, Morosphaeriaceae, Sulcatisporaceae and Trematosphaeriaceae) were included, with *Phaeosphaeriaoryzae* (CBS 110110) and *Phaeosphaeriopsisglaucopunctata* (MFLUCC 13-0265) (Phaeosphaeriaceae) as the outgroup. Primarily, phylogenetic analysis of the concatenated LSU, SSU and ITS sequence dataset was conducted, based on ML and compared with phylogenetic analysis of the concatenated ITS, LSU, SSU and *tef1-α* sequence dataset. Phylogenetic analysis, based on the concatenated LSU, SSU and ITS sequence dataset, showed a similar topology with the concatenated ITS, LSU, SSU and *tef1-α* sequence dataset and were not significantly different (data not shown). Hence, multigene phylogenetic analysis of the concatenated ITS, LSU, SSU and *tef1-α* gene regions was selected to represent the phylogenetic relationships of *Trichobotryssinensis* sp. nov. with other closely-related species in Dictyosporiaceae. The aligned dataset contained 3729 characters, including gaps. Phylogenetic relationships were inferred by conducting analyses using both ML and BI methods. The best-scoring RAxML tree was selected to represent the relationships amongst taxa, with a final likelihood value of -28366.415110 (Fig. 2). The matrix contained 1566 distinct alignment patterns, with a 39.19% proportion of gaps and completely undetermined characters. The estimated base frequencies of A = 0.239629, C = 0.244575, G = 0.269426, T = 0.246371; substitution rates AC = 1.123110, AG = 2.634717, AT = 1.787337, CG = 0.836519, CT = 6.160493, GT = 1.000000; gamma distribution shape parameter α = 0.461486; Tree-Length = 3.107341. For BI analysis, GTR + I + G was selected as the best-fit model by AIC in MrModelTest for each gene (ITS, LSU, SSU and *tef1-α*). Six simultaneous Markov chains were run for 4,085,000 generations, resulting in 40,851 total trees. The first 10% of trees were discarded as the burn-in phase of the analyses and the remaining trees were used for calculating posterior probabilities in the majority rule consensus tree, of which the final average standard deviation of split frequencies at the end of total MCMC generations was 0.009998.

Multigene phylogenetic analyses of the concatenated ITS, LSU, SSU and *tef1-α* demonstrated that all representative families formed well-resolved clades in the present study. Our new isolate grouped with two unnamed *Gregarithecium* sp. (strains GMB1217 and MFLUCC 13-0853), with high support in ML and BI analyses (99% ML, 100 PP; Fig. 2) and clustered with *Trichobotryseffusus* (strains 1179, HNNUZCJ-94, FS524, SYSU-MS4729 and DFFSCS021) with high support (100% ML, 1.00 PP; Fig. 2) in Dictyosporiaceae. *Gregarithecium* sp. (strains GMB1217 and MFLUCC 13-0853) is unpublished and showed to be conspecific with our new isolate. Therefore, our new isolate is introduced as *Trichobotryssinensis*, based on phylogenetic evidence coupled with morphological characteristics. *Trichobotrys* formed a highly-supported subclade with *Gregarithecium* (99% ML, 1.00 PP; Fig. 2) in the present study. However, these two genera are represented by different morphs. Therefore, the congeneric status of these two genera is doubtful in the study pending future study.

#### ﻿Analysis 3

**Figure 3.**
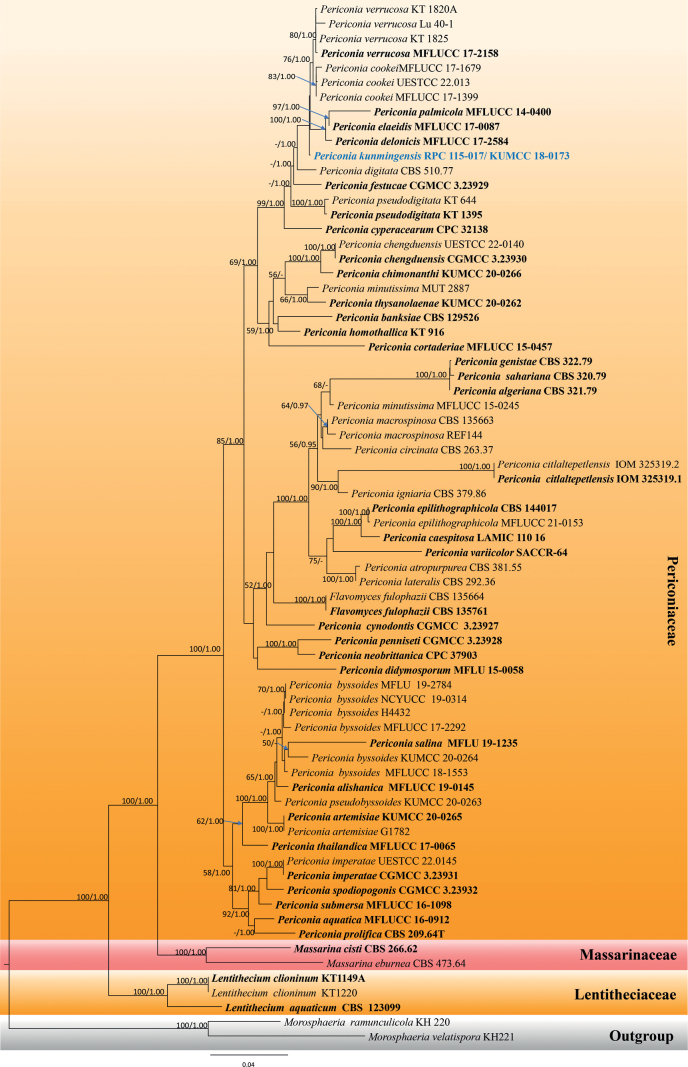
Phylogram of the best-scoring ML consensus tree of taxa in Periconiaceae and the closely-related families Lentitheciaceae and Massarinaceae. The new isolate is indicated in blue. Isolates from type materials are in bold. The ML ultrafast bootstrap and Bayesian PP values greater than 50% and 0.95 are shown at the nodes.

The *Periconia* species tree was constructed using sequence data from ITS, LSU, SSU and *tef1-α* of all taxa in Periconiaceae and other related families (viz. Lentitheciaceae, and Massarinaceae). A total of 71 strains were included, with *Morosphaeriaramunculicola* (KH 220) and *M.velatispora* (KH 221) as the outgroup. The aligned dataset contained 3646 characters, including gaps. The best-scoring RAxML tree was selected to represent the relationships amongst taxa, with a final likelihood value of -19141.848334 (Fig. 3). The matrix contained 1265 distinct alignment patterns, with a 32.87% proportion of gaps and completely undetermined characters. The estimated base frequencies of A = 0.239678, C = 0.253426, G = 0.268914, T = 0.237981; substitution rates AC = 1.751555, AG = 3.051838, AT = 1.900841, CG = 1.359429, CT = 9.411951, GT = 1.000000; gamma distribution shape parameter α = 0.505775; Tree-Length = 1.483987. For BI analysis, GTR + I + G was selected as the best-fit model by AIC in MrModelTest for each gene (ITS, LSU, SSU and *tef1-α*). Six simultaneous Markov chains were run for 555,000 generations, resulting in 5551 total trees. The first 10% of trees were discarded as the burn-in phase of the analyses and the remaining trees were used for calculating posterior probabilities in the majority rule consensus tree, of which the final average standard deviation of split frequencies at the end of total MCMC generations was 0.009941.

Multigene phylogenetic analyses demonstrated that the new species *Periconiakunmingensis* (KUMCC 18-0173) formed a distinct lineage and clustered with the clade containing *P.cookei* (MFLUCC 17-1679, MFLUCC 17-1399 and UESTCC 22.013), *P.delonicis*, (MFLUCC 17-2584), *P.elaeidis* (MFLUCC 17-0087), *P.palmicola* (MFLUCC 14-0400) and *P.verrucosa* (MFLUCC 17-2158, Lu40-1, KT1820A and KT1825), with strong statistical support (100% ML, 1.00 PP; Fig. 3).

## ﻿Taxonomy

### ﻿ Bambusicolaceae

Taxon classificationFungiPleosporalesBambusicolaceae

D.Q. Dai & K.D. Hyde, Fungal Diversity 63: 49 (2013)

896D3838-ABD8-5F94-B86C-28C0447E2920

Index Fungorum: IF804293

#### Notes.

Bambusicolaceae was first introduced by Hyde et al. (2013) to accommodate *Bambusicola* with *B.massarinia* being the type species. Subsequently, another three genera were accommodated in this family viz. *Corylicola* (Wijesinghe et al. 2020), *Leucaenicola* (Jayasiri et al. 2019) and *Palmiascoma* (Liu et al. 2015). Species of these genera have been reported from various hosts, such as *Camellia*, *Corylus*, *Eucalyptus*, Fagaceae sp., *Leucaena*, *Osmanthus* and palm and so far, found distributed in China (Sichuan and Yunnan), Italy and Thailand (Liu et al. 2015; Jayasiri et al. 2019; Ariyawansa et al. 2020a, b; Hongsanan et al. 2020; Wijesinghe et al. 2020; Monkai et al. 2021). Members of Bambusicolaceae are mainly saprobes; however, Ariyawansa et al. (2020a, b) reported that species of *Leucaenicola* associated with leaf spot diseases on *Camellia* and *Osmanthus* in Taiwan (China). Bambusicolaceae is a well-studied family, based on morphological characteristics of sexual-asexual morphs and multigene phylogenetic evidence. Recent taxonomic treatment carried out by Hongsanan et al. (2020) revealed that the family belongs to the suborder Massarineae, order Pleosporales of Dothideomycetes, comprising four genera and 25 species (http://www.indexfungorum.org; accessed on 25 May 2023).

### ﻿ Bambusicola

Taxon classificationFungiPleosporalesBambusicolaceae

D.Q. Dai & K.D. Hyde, Cryptog. Mycol. 33(3): 367 (2012)

42DF2EDA-6DEB-58CF-9B43-8CDD5E37978A

Index Fungorum: IF801041

#### Notes.

*Bambusicola* was introduced by Dai et al. (2012) to accommodate four saprobic species associated with bamboo, namely *B.bambusae*, *B.irregulispora*, *B.massarinia* and *B.splendida*. Subsequently, many species were included in the genus which were mainly known as saprobes on different bamboos in terrestrial habitats (Dai et al. 2012, 2015, 2017; Thambugala et al. 2017; Monkai et al. 2021; Phukhamsakda et al. 2022; Yu et al. 2022). However, *B.sichuanensis* and *B.subthailandica* were reported as parasites on *Phyllostachysheteroclada* (Yang et al. 2019). While *B.aquatica* was reported as a saprobe submerged in freshwater (Dong et al. 2020) and *B.ficuum* was reported on dead twigs of *Ficus* (Brahmanage et al. 2020). *Bambusicola* is morphologically well-studied and appear pleomorphic. Besides, its phylogenetic affinities have been well-clarified, based on multigene phylogenetic evidence (e.g. *B.didymospora*, *B.massarinia*, *B.triseptatispora*) (Dai et al. 2012, 2017). Currently, there are 17 species accepted in the genus, mostly distributed in the Sichuan and Yunnan Provinces of China and Thailand (http://www.indexfungorum.org; accessed on 25 May 2023). In the present study, we introduce a novel species *B.hongheensis* which was collected from dead bamboo culms in Yunnan, China.

### ﻿ Bambusicola hongheensis

Taxon classificationFungiPleosporalesBambusicolaceae

Phookamsak, Bhat & Hongsanan sp. nov.

EBE6B320-314F-5BB9-9DA5-839369AD3EE3

Index Fungorum: IF900830

Fig. 4

#### Etymology.

The specific epithet “*hongheensis*” refers to the locality, Honghe Hani and Yi Autonomous Prefecture (Yunnan, China), where the holotype was collected.

**Figure 4.**
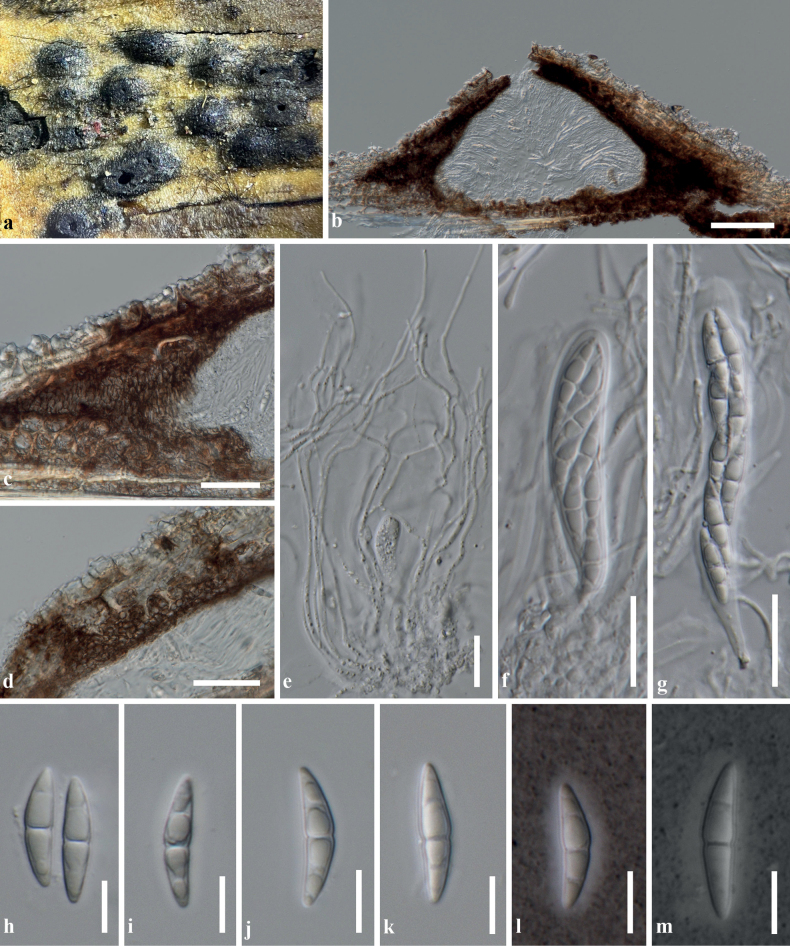
*Bambusicolahongheensis* (KUN-HKAS 129042, holotype) **A** the appearance of ascomata on the host surface **B** vertical section of an ascoma **C, D** peridia **E** pseudoparaphyses **F, G** asci embedded in pseudoparaphyses **H–K** ascospores **L, M** ascospores stained in India Ink show a thin mucilaginous sheath surrounding ascospores. Scale bars: 100 μm (**B**); 20 μm (**C–G**); 10 μm (**H–M**).

#### Holotype.

KUN-HKAS 129042.

#### Description.

Saprobic on dead culm of bamboo in terrestrial habitats, visible as black, shiny, gnarled on the host surface. ***Sexual morph*: *Ascomata*** 225–350 μm high, 340–590 μm diam., scattered, sometimes forming stroma with a clustered 1–3 ascomata, gregarious, semi-immersed, raised, becoming superficial, dark brown, dome-shaped to subconical or subglobose, glabrous, coriaceous, ostiolate with inconspicuous papilla. ***Peridium*** 40–80(–130) μm wide at sides towards the apex, 10–25 μm wide at the base, composed of several layers of small, dark brown pseudoparenchymatous cells, outer layer fused with host cells, arranged in *textura angularis* to *textura globulosa*, inner layer composed of 1–3 strata of flattened cells, of *textura globulosa* to *textura prismatica*, with thick, palisade-like cells at the sides. ***Hamathecium*** composed of 1–3 μm wide, filiform, dense, septate, branched, pseudoparaphyses, anastomosed between and above the asci, embedded in a gelatinous matrix. ***Asci*** (58–)70–90(–105)(–119) × 12–15(–17) μm (*x̄* = 80.5 × 13.5 μm, SD = ± 13.2 × 1.8, n = 25), 8-spored, bitunicate, fissitunicate, cylindrical-clavate, shortly pedicellate, apically rounded with well-developed ocular chamber. ***Ascospores*** 22–26(–30) × 4.5–7 μm (*x̄* = 24.6 × 5.4 μm, SD = ± 2.3 × 0.5, n = 30), overlapping 1–3-seriate, hyaline, fusiform, slightly curved, 1-septate, occasionally 2–3-septate, slightly constricted at the septum, the upper cell slightly larger than the lower cell, smooth-walled, surrounded by a thin, indistinct, mucilaginous sheath. ***Asexual morph***: Undetermined.

#### Distribution.

China (Yunnan).

#### Specimen examined.

China. Yunnan Province: Honghe Hani and Yi Autonomous Prefecture, Honghe County, rice terraces, on dead culm of bamboo, 26 Jan 2021, R. Phookamsak BN06 (KUN-HKAS 129042, **holotype**). **Notes**: As the axenic culture is not active, the sequences of SSU and *rpb2* were obtained from genomic DNA extracted from ascomata and dried culture.

#### Notes.

Based on the NCBI nucleotide BLAST search of ITS sequence, *Bambusicolahongheensis* (KUN-HKAS 129042) has the closest match with *B.triseptatispora* (MFLUCC 11-0166, ex-type strain) with 98.71% similarity (Identities = 535/542 with no gap) and is similar to *B.loculata* (MFLU 15-0056, ex-type strain) with 98.69% similarity (Identities = 528/535 with 1 gap) and *B.splendida* (MFLUCC 11-0611) with 98.25% similarity (Identities = 392/399 with no gap). The NCBI nucleotide BLAST search of LSU sequence indicated that *B.hongheensis* has the closest match with *B.triseptatispora* (MFLUCC 11-0166, ex-type strain) and *B.didymospora* (MFLUCC 10-0557, ex-type strain) with 100% similarity (Identities = 802/802 with no gap) and is similar to *B.loculata* (MFLU 15-0056, ex-type strain) with 99.75% similarity (Identities = 813/815 with 2 gaps) and *B.nanensis* (MFLUCC 21-0063, ex-type strain) with 99.49% similarity (Identities = 785/789 with no gap). The NCBI nucleotide BLAST search of *rpb2* sequence indicated that *B.hongheensis* has the closest match with *B.loculata* (MFLU 15-0056, ex-type strain) with 99.90% similarity (Identities = 1042/1043 with no gaps) and is also similar to *B.triseptatispora* (MFLUCC 11-0166, ex-type strain) with 97.92% similarity (Identities = 990/1011 with no gap) and *B.massarinia* (voucher MFLU 11-0389) with 93.57% similarity (Identities = 975/1042 with 4 gaps).

Phylogenetic analyses of a concatenated ITS, LSU, *rpb2*, SSU and *tef1-α* sequence dataset demonstrated that *Bambusicolahongheensis* formed a separate branch (85% ML, 1.00 PP; Fig. 1), and clustered with *B.loculata* and *B.triseptatispora* with high support (100% ML, 1.00 PP; Fig. 1) and also clustered with the generic type of *Bambusicola*, *B.massarinia* with significant support (73% ML, 0.99 PP; Fig. 1). A nucleotide pairwise comparison of ITS sequence indicated that *B.hongheensis* differs from *B.triseptatispora* in 35/600 bp (5.83%), differs from *B.loculata* in 16/547 bp (2.92%) and differs from *B.massarinia* in 72/608 bp (11.84%). Whereas the nucleotide pairwise comparison of LSU sequence indicated that *B.hongheensis* is consistent with *B.triseptatispora* (0/802 bp) and *B.loculata* (1/816 bp), but differs from *B.massarinia* in 7/803 bp (0.87%). Furthermore, the nucleotide pairwise comparison of *rpb2* sequence indicated *B.hongheensis* is not significantly different from *B.loculata* (1/1043 bp), but differs from *B.triseptatispora* in 21/1012 bp (2.07%) and differs from *B.massarinia* in 68/1042 bp (6.52%).

Morphologically, *Bambusicolahongheensis* resembles *B.loculata* and *B.triseptatispora* in terms of the size range of ascomata, asci and ascospores. However, *B.hongheensis* has comparatively smaller ascomata (340–590 μm diam. of *B.hongheensis* vs. 350–600 μm diam. of *B.loculata* vs. 470–730 μm diam. of *B.triseptatispora*), shorter and wider asci ((58–)70–90(–105)(–119) × 12–15(–17) μm vs. 80–105 × 8–13 μm vs. (78–)80–100(−110) × 10–12(−14) μm, respectively) and sharing the size range of ascospores (22–26(–30) × 4.5–7 μm vs. 22–26.5 × 5–6 μm vs. (25–)26–30(−31) × 4–6 μm, respectively). The ascospores of *B.hongheensis* are typically hyaline, 1-septate, whereas *B.triseptatispora* has hyaline to pale brown and 3-septate ascospores (Dai et al. 2017). Distinguishing *B.loculata* from *B.hongheensis*, based on morphological characteristics alone is challenging, but *B.loculata* can be differentiated by its larger ascomata and asci (Dai et al. 2015). However, a clear differentiation is achieved through phylogenetic evidence (Fig. 2) and nucleotide pairwise comparison of ITS gene region (2.92% difference).

### ﻿ Dictyosporiaceae

Taxon classificationFungiPleosporalesDictyosporiaceae

Boonmee & K.D. Hyde, in Boonmee et al., Fungal Diversity: 10.1007/s13225-016-0363-z, [7] (2016)

DEEF0297-BCD6-5176-A27C-B85343343124

Index Fungorum: IF551574

#### Notes.

Dictyosporiaceae was introduced by Boonmee et al. (2016) to initially accommodate ten genera that were mainly represented by the hyphomycetous asexual morph, forming cheiroid, digitate, palmate and/or dictyosporous conidia. The sexual morph is scarcely known for this family, of which species of genera *Dictyosporium*, *Gregarithecium*, *Immotthia*, *Pseudocoleophoma*, *Sajamaea* and *Verrucoccum* have been represented as the sexual morph (Boonmee et al. 2016; Piątek et al. 2020; Atienza et al. 2021; Jiang et al. 2021a). Members of Dictyosporiaceae are morphologically diverse in various ecological niches, commonly known as saprobes on plant litter in terrestrial and freshwater habitats (Tanaka et al. 2015; Boonmee et al. 2016; Li et al. 2017; Crous et al. 2019; Rajeshkumar et al. 2021; Tian et al. 2022; Tennakoon et al. 2023). Besides, some genera were known as fungicolous (hyperparasites and mycoparasites) and lichenicolous fungi as well as inhabiting soil and herbivore dung (Iturrieta-González et al. 2018; Piątek et al. 2020; Atienza et al. 2021; Jiang et al. 2021a). An updated taxonomic description of Dictyosporiaceae was provided by Hongsanan et al. (2020) who listed 15 genera in this family, while Wijayawardene et al. (2022b) listed 17 genera in Dictyosporiaceae. Tennakoon et al. (2023) provided a backbone tree of Dictyosporiaceae and currently listed 20 genera in this family, namely *Aquadictyospora*, *Aquaticheirospora*, *Cheiro­sporium*, *Dendryphiella*, *Dictyocheirospora*, *Dictyopalmispora*, *Dictyo­sporium*, *Digito­desmium*, *Gregarithecium*, *Immotthia*, *Jalapriya*, *Neodendryphiella*, *Neodigito­desmium*, *Pseudocoleophoma*, *Pseudoconiothyrium*, *Pseudocyclo­thyriella*, *Pseudodictyosporium*, *Sajamaea*, *Verrucoccum* and *Vikalpa*.

### ﻿ Trichobotrys

Taxon classificationFungiPleosporalesDictyosporiaceae

Penz. & Sacc., Malpighia 15(7–9): 245 (1902) [1901]

2FA25B85-ED64-579E-8EA3-600AA01538B2

Index Fungorum: IF10275

#### Notes.

*Trichobotrys* was introduced by Penzig and Saccardo (1902) to accommodate the type species *T.pannosus* [as ‘*pannosa*’]. The genus is scarcely known and only five species are available in Index Fungorum (http://www.indexfungorum.org; accessed on 25 May 2023), of which only *T.effusus* [as ‘*effusa*’] has molecular data available in GenBank. The genus is known as only a hyphomycetous asexual morph and is characterised by dark brown to black, effuse to velvety colonies, partly immersed to superficial mycelium, non-stromatic, macronematous, mononematous, dark brown to reddish-brown, verruculose or echinulate conidiophores, bearing short, smooth, fertile, often unciform lateral branches, with sterile, setiform apex, polyblastic, integrated, terminal or discrete, determinate, ellipsoidal, spherical or subspherical conidiogenous cells and catenated, in branched acropetal chains, spherical, brown, aseptate, verruculose or minutely echinulate conidia (Ellis 1971; D’Souza and Bhat 2001). The taxonomic classification of the genus is doubtful due to the lack of molecular phylogeny. Recently, Wijayawardene et al. (2022b) treated *Trichobotrys* as Ascomycota genus *incertae sedis*, pending future study. In the present study, the novel species, *T.sinensis* is introduced and the phylogenetic analyses demonstrated the genus affinity in Dictyosporiaceae.

### ﻿ Trichobotrys sinensis

Taxon classificationFungiPleosporalesDictyosporiaceae

Phookamsak, Bhat & Hongsanan sp. nov.

49C20070-18C2-5650-87C4-419C204D1167

Index Fungorum: IF900832

Fig. 5

#### Etymology.

The specific epithet “*sinensis*” refers to the country, China, where the holotype was collected.

**Figure 5.**
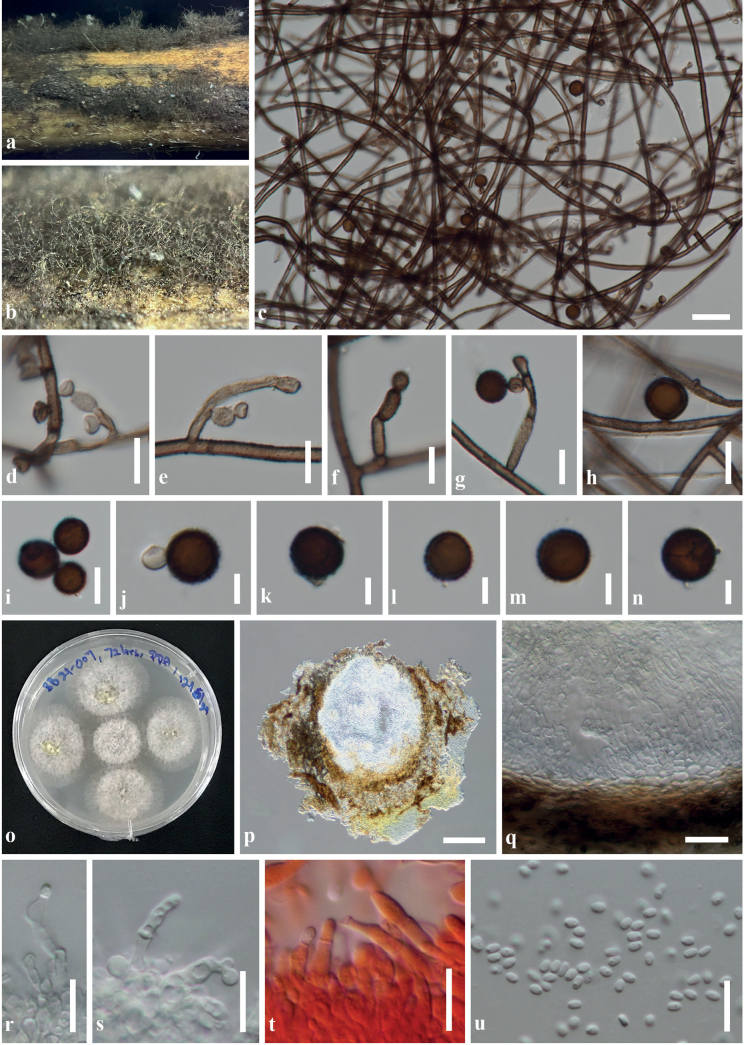
*Trichobotryssinensis* (KUN-HKAS 129041, holotype) **A, B** the appearance of colonies on the host surface **C** mycelium **D–H** conidiophores bearing conidiogenous cells and conidia **I** conidia in a short acropetal chain **J–N** conidia **O** culture characteristics on PDA**P** conidioma forming on PDA after eight weeks **Q** pycnidial wall **R–T** conidiogenous cells (note: **T** = stained in Congo red) **U** conidia. Scale bars: 100 μm (**P**); 50 μm (**C**); 10 μm (**D–H, Q–U**); 5 μm (**J–N**).

#### Holotype.

KUN-HKAS 129041.

#### Description.

Saprobic on dead culm of *Brachiariamutica*, submerged in a small stream. ***Sexual morph***: Undetermined. ***Asexual morph*: *Colonies*** dull, black, effuse, visible as hairy fluffy on the host. ***Mycelia*** up to 1 mm long, 2–4 µm wide, superficial, composed of brown to dark brown, branched, septate, thick-walled, echinulate hyphae. ***Conidiophores*** (9–)15–40(–70) × 2–4 µm (*x̄* = 26.9 × 3.3 μm, n = 30), sometimes reduced to conidiogenous cells, macronematous, mononematous, straight or flexuous, brown to dark brown, septate, verruculose or echinulate, bearing short, lateral, unciform, fertile branches, with setiform apex. ***Conidiogenous cells*** 1–3.5 × 2.5–5 µm (*x̄* = 2.1 × 2.5 μm, n = 30), polyblastic, subhyaline to pale brown, ellipsoidal or hemispherical (2.5–5 × 3.5–6 µm), intercalary or terminal, integrated or discrete, sometimes denticulate on branches. ***Conidia*** 7–11 × 8–12 µm (*x̄* = 10 × 10 μm, n = 30) simple, solitary, brown to dark brown, spherical, aseptate, verruculose; sometimes in short acropetal chains. *In vitro****Conidiomata*** 280–470 µm high, 280–570 µm diam., black, pycnidial, solitary or clustered in a small group (2–4-loculate), scattered to gregarious, globose to subglobose, glabrous, covered by brown to dark brown mycelium, becoming a packed pycnidial wall, ostiolate, with inconspicuous, minute papilla. ***Pycnidial wall*** 20–35 µm wide, thick-walled of unequal thickness, thicker at the base, composed of multi-layered, dark brown to black pseudoparenchymatous cells, outer layers composed of *textura intricata*, inner layers composed of flattened cells of *textura angularis* to *textura prismatica*. ***Conidiophores*** reduced to conidiogenous cells. ***Conidiogenous cells*** (6.5–)10–16(–25) × 2–4.5 µm (*x̄* = 13.4 × 3.2 μm, n = 30), holoblastic to phialidic, hyaline, cylindrical to subcylindrical, terminal or intercalary, septate, smooth-walled, with distinct collarette. ***Conidia*** 2–3 × 1.5–2.5 µm (*x̄* = 2.8 × 2 μm, n = 30) hyaline, ellipsoidal to ovoid, aseptate, smooth-walled, with a guttulate.

#### Culture characteristics.

Colonies on PDA reaching 25–28 mm diam. after two weeks at room temperature (20–27 °C), medium dense, circular, surface smooth with an entire edge, flattened, slightly raised, fairly fluffy to feathery; from above, initially white, with cream conidial masses, becoming white to cream at the margin, pale yellowish towards the centre with age; from below, white at the margin, dark grey to black towards the centre; pigmentation not produced in PDA. Sporulation in PDA after two weeks, initially visible as cream conidial masses, later forming black conidiomata with hyaline to cream conidial masses on colonies.

#### Distribution.

China (Yunnan).

#### Specimen examined.

China. Yunnan Province: Xishuangbanna Dai Autonomous Prefecture, Mengla County, Bubeng, 21°36'30.13"N, 101°35'52.54"E, 664 + 5 m a.s.l., on culms of *Brachiariamutica* submerged in a freshwater stream, 27 Apr 2021, R. Phookamsak BB21-007 (KUN-HKAS 129041, **holotype**), ex-type living culture, RPC 21-007 = KUNCC 23-14554.

#### Notes.

Based on NCBI nucleotide BLAST search of ITS sequence, the closest hit of *Trichobotryssinensis* (RPC 21-007/ KUNCC 23-14554) is *Gregarithecium* sp. DQD-2016a strain MFLUCC 13-0853 with 99.03% similarity (Identities = 508/513 with 2 gaps) and is similar to *Trichobotryseffusus* [as ‘*effusa*’] isolate 1179 (93.51% similarity, Identities = 504/539 with 13 gaps), *T.effusus* [as ‘*effusa*’] strain FS522 (93.35% similarity, Identities = 477/511 with 12 gaps) and *T.effusus* [as ‘*effusa*’] isolate HNNUZCJ-94 (93.08% similarity, Identities = 471/506 with 16 gaps). In LSU nucleotide BLAST search, the closest hit of *T.sinensis* (RPC 21-007/ KUNCC 23-14554) is *Gregarithecium* sp. DQD-2016a strain MFLUCC 13-0853 with 99.88% similarity (Identities = 848/849 with 1 gap) and is similar to *Gregarithecium* sp. isolate L13E (99.40% similarity, Identities = 830/835 with 3 gaps) and *G.curvisporum* HHUF 30134 (97.74% similarity, Identities = 822/841 with 5 gaps).

Multigene phylogenetic analyses of a concatenated ITS, LSU, SSU and *tef1-α* sequence dataset demonstrated that *Trichobotryssinensis* (RPC 21-007/ KUNCC 23-14554) shared a branch length with *Gregarithecium* sp. DQD-2016a strain MFLUCC 13-0853 and *Gregarithecium* sp. isolate GMB 1217 and clustered with the clade of *T.effusus* (Fig. 2). However, *Gregarithecium* sp. DQD-2016a strain MFLUCC 13-0853 and *Gregarithecium* sp. isolate GMB 1217 are unpublished strains. Hence, *Trichobotryssinensis* (RPC 21-007/ KUNCC 23-14554) is introduced herein as a new species and *Gregarithecium* sp. (strains MFLUCC 13-0853 and isolate GMB 1217) is re-identified as *T.sinensis* to avoid misidentification. Morphologically, *T.sinensis* (RPC 21-007/ KUNCC 23-14554) is typical of *Trichobotrys*, but can be distinguished from *T.effusus*, *T.pannosus*, *T.ramosus* and *T.trechisporus* in having larger conidia (2 µm diam. of *T.effusus* vs. 4 µm diam. of *T.pannosus* vs. 3–5 µm diam. of *T.ramosus* vs. 5 × 3 µm or 4 µm diam. of *T.trechisporus*) (Berkeley and Broome 1873; Penzig and Saccardo 1902; Petch 1917; Ellis 1971; D’Souza and Bhat 2001).

### ﻿ Periconiaceae

Taxon classificationFungiPleosporalesPericoniaceae

Nann., Repert. mic. uomo: 482 (1934)

D0747C5C-7962-5E6C-8223-63898767E14E

Index Fungorum: IF81124

#### Notes.

Periconiaceae was resurrected by Tanaka et al. (2015) who provided an updated taxonomic treatment and placed the family in the suborder Massarineae (Pleosporales). Tanaka et al. (2015) accepted four genera namely, *Periconia* (Tode 1791), *Noosia* (Crous et al. 2011), *Bambusistroma* (Adamčík et al. 2015) and *Flavomyces* (Knapp et al. 2015), as well as included *Sporidesmiumtengii* in the Periconiaceae. Yang et al. (2022b) re-circumscribed genera *Bambusistroma*, *Noosia* and *Periconia*, based on type studies compared with their new findings. Hence, Yang et al. (2022b) treated *Bambusistroma* and *Noosia* as synonyms of *Periconia* due to morphological resemblances and phylogenetic evidence, while the generic status of *Flavomyces* is doubted pending further studies.

### ﻿ Periconia

Taxon classificationFungiPleosporalesPericoniaceae

Tode, Fung. mecklenb. sel. (Lüneburg) 2: 2 (1791)

900300E7-F98F-5EFA-AF57-08869540F86F

Index Fungorum: IF9263

#### Notes.

*Periconia* was established by Tode (1791) to accommodate dematiaceous hyphomycetes that were unique in forming macronematous, mononematous, branched, septate, pigmented conidiophores, bearing spherical conidial heads that produced globose to ellipsoidal, aseptate, verruculose to echinulate, pigmented conidia (Tanaka et al. 2015; Hongsanan et al. 2020; Yang et al. 2022b). Species of *Periconia* are typically known by their asexual morph; only a few species have been reported with their sexual morph (Tanaka et al. 2015; Hongsanan et al. 2020; Yang et al. 2022b). *Periconia* species have been commonly reported as saprobes occurring on various host substrates in terrestrial and aquatic habitats worldwide. However, some species have been reported as endophytes, plant pathogens (e.g. *P.circinata*, *P.digitata* and *P.macrospinosa*) and human pathogens, as well as producing economically-important bioactive compounds (Sarkar et al. 2019; Gunasekaran et al. 2021; Hongsanan et al. 2020; Samarakoon et al. 2021; Azhari and Supratman 2021; Yang et al. 2022b; Su et al. 2023). Even though over 200 species of *Periconia* were listed in Index Fungorum (http://www.indexfungorum.org; accessed on 25 May 2023), less than half of a quarter have molecular data to clarify phylogenetic placement. Of these, the type species of *Periconia*, *P.lichenoides*, also lacks molecular data. This suggests that there is a huge research gap in the taxonomic classification of the genus *Periconia*. In the present study, we follow the latest taxonomic treatment of Yang et al. (2022b) and Su et al. (2023) and the new species *Periconiakunmingensis* occurring on fern, is introduced.

### ﻿ Periconia kunmingensis

Taxon classificationFungiPleosporalesPericoniaceae

Phookamsak & Hongsanan sp. nov.

4E5988EE-BB87-5946-B836-46DC18DBD7C7

Index Fungorum: IF900833

Fig. 6

#### Etymology.

The specific epithet “*kunmingensis*” refers to the Kunming Institute of Botany, Kunming, Yunnan, China, where the holotype was collected.

**Figure 6.**
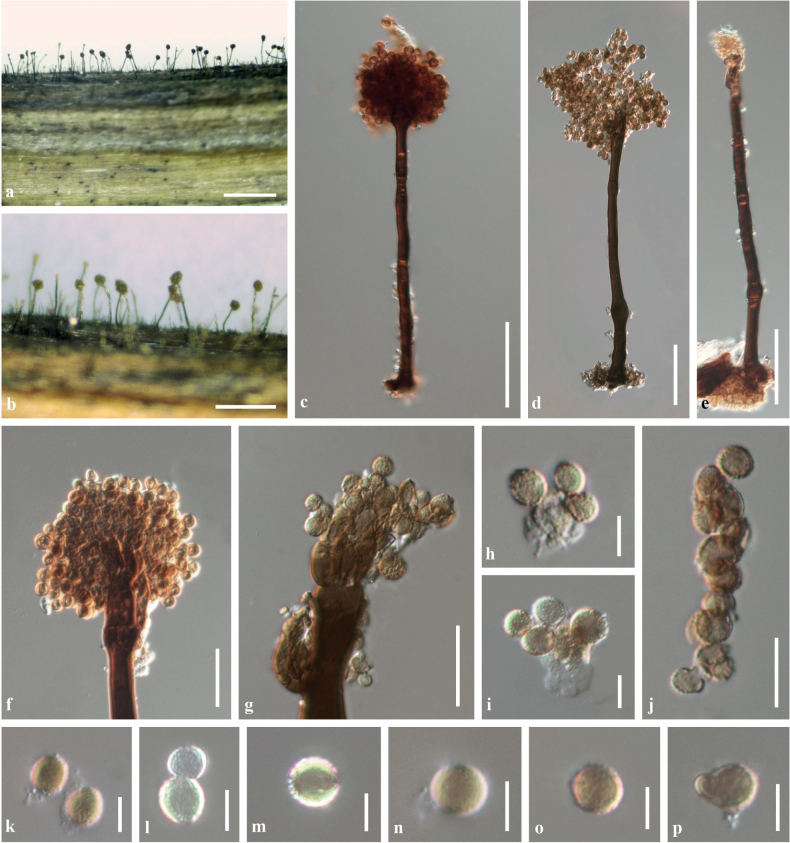
*Periconiakunmingensis* (KUN-HKAS102239, holotype) **A, B** the appearance of fungal colonies on host substrate **C–E** conidiophores **F, G** closed-up conidiophores with spherical heads **H, I** conidiogenous cells bearing conidia **J** conidia catenate in acropetal short chain **K–P** conidia. Scale bars: 500 µm (**A, B**); 50 µm (**C–E**); 20 µm (**F**, **G**); 10 µm (**J**); 5 µm (**H, I, K–P**).

#### Holotype.

KUN-HKAS 102239.

#### Description.

Saprobic on dead, standing rachis of a fern. ***Sexual morph***: Undetermined. ***Asexual morph*: *Colonies*** on the substrates superficial, numerous, effuse, brown to dark brown, floccose. ***Mycelia*** 6–7 μm wide, partly superficial, composed of septate, branched, dark brown hyphae. ***Conidiophores*** 100–260 μm long, 7–12 μm diam., macronematous, mononematous, solitary, dark brown, 3–5-septate, unbranched below, branched only at the apex, erect, straight or slightly flexuous, sometimes swollen near the base, with 1–2 spherical guttules in each cell, forming a spherical head at the tip. ***Conidiogenous cells*** (4–)5–8(–10) × 2.5–5(–6) μm (*x̄* = 6.4 × 4 μm, n = 30) mono- to polyblastic, terminal, discrete, subspherical to fusiform, subhyaline to pale brown, verruculose. ***Conidia*** 4.5–7(–9) × 4–7(–8) μm (*x̄* = 6 × 5.9 μm, n = 50), solitary to catenate, in acropetal short chains, subglobose to globose, subhyaline to pale brown, aseptate, minutely verruculose to short-spinulose.

#### Culture characteristics.

Colonies on PDA reaching 23–25 mm diam. after two weeks at room temperature (20–30 °C). Colony dense, circular, flattened, slightly raised, surface smooth, edge fimbriate, velvety, with fairly fluffy at the margin; colony from above, white to white-grey, separated from the centre by greenish-grey radiating near the margin; colony from below, pale yellowish to cream at the margin, deep green near the margin, with dark green concentric ring, separating the margin from greenish-grey to dark green centre; slightly produced light yellowish pigment tinted agar.

#### Distribution.

China (Yunnan).

#### Specimen examined.

China. Yunnan Province: Kunming, Kunming Institute of Botany, on dead, standing rachis of a fern, 23 Sep 2016, R. Phookamsak KIB004 (KUN-HKAS 102239, **holotype**), ex-type living culture RPC 15-017 = KUMCC 18-0173 = MFLUCC 18-0679. Addition GenBank no: *rpb2* = OR547996.

#### Notes.

Based on the NCBI nucleotide BLAST search of ITS sequence, the closest hits of *Periconiakunmingensis* are *Periconia* sp. strain 8R5B1-3 and *Periconia* sp. isolate LS77 with 99.80% similarity (Identities = 507/508 and 498/499 with no gap, respectively) and is similar to *P.verrucosa* isolate HNNU0545 with 99.60% similarity (Identities = 502/504 with 1 gap), *Periconia* sp. strain MFLUCC 17-0087 with 99.59% similarity (Identities = 482/484 with 1 gap) and *P.elaeidis* isolate PT49 with 99.57% similarity (Identities = 464/466 with 1 gap). In the LSU nucleotide BLAST search, *P.kunmingensis* is similar to *P.verrucosa* isolate MFLUCC 17-2158 (Identities = 847/847 with no gap), *Periconia* sp. KT 1825 (Identities = 843/843 with no gap), *P.elaeidis* strain GZCC19-0435 (Identities = 842/842 with no gap), *P.cookei* strain IHEM:28143 (Identities = 826/826 with no gap), Pleosporales sp. A1039 (Identities = 815/815 with no gap) and *P.verrucosa* isolate w232_2 (Identities = 812/812 with no gap), isolate Lu53_1 (Identities = 807/807 with no gap) and isolate Lu98_2 (Identities = 796/796 with no gap), with 100% similarities. In the *tef1-α* nucleotide BLAST search, the closest hit of *P.kunmingensis* is *Periconia* sp. KT 1820A (Identities = 745/747 with no gap) and *P.delonicis* voucher MFLU 20-0696 (Identities = 736/738 with no gap) with 99.73% similarity. *Periconiakunmingensis* is also similar to *P.delonicis* strain MFLUCC 17-2584 and *P.verrucosa* isolate MFLUCC 17-2158 with 99.60% similarity (Identities = 744/747 with no gap).

Phylogenetic analyses of the concatenated ITS, LSU, SSU and *tef1-α* sequence data showed that *Periconiakunmingensis* formed a distinct branch basally to *P.verrucosa*, *P.cookei*, *P.palmicola*, *P.elaeidis* and *P.delonicis*, respectively (Fig. 3). The ITS nucleotide pairwise comparison indicated that *P.kunmingensis* differs from *P.verrucosa* (MFLUCC 17–2158, ex-type strain) in 3/512 bp (0.59%), differs from *P.cookei* in 2/465 bp (0.43%) of MFLUCC 17–1399 and 3/465 bp (0.65%) of UESTCC 22.0134 and differs from *P.elaeidis* (MFLUCC 17–0087, ex-type strain) in 14/518 bp (2.70%). The *rpb2* nucleotide pairwise comparison indicated that *P.kunmingensis* differs from *P.verrucosa* (UESTCC 22.0136) in 35/849 bp (4.12%), differs from *P.cookei* (UESTCC 22.0134) in 30/819 bp (3.66%) and differs from *P.delonicis* (MFLUCC 17–2584, ex-type strain) in 54/1073 bp (5.03%). The *tef1-α* nucleotide pairwise comparison indicated that *P.kunmingensis* differs from *P.verrucosa* (MFLUCC 17–2158, ex-type strain) in 108/929 bp (11.63%), differs from *P.cookei* in 4/736 bp (0.54%) of MFLUCC 17-1399 and 107/906 bp (11.81%) of MFLUCC 17-1679, differs from *P.palmicola* (MFLUCC 14-0400, ex-type strain) in 19/991 bp (1.92%) and differs from *P.delonicis* (MFLUCC 17–2584, ex-type strain) in 105/987 bp (10.64%).

Distinguishing *Periconiakunmingensis* from other *Periconia* species, based on morphological features alone, presents challenges. However, differentiation can be achieved by considering variations in the sizes of conidiophores, conidiogenous cells and conidia, as well as the number of conidiophores originating from the stromatic, swollen part of the conidiophores, septation characteristics and the occurrence and origin of the host. A comprehensive morphological comparison is provided in Table 4.

**Table 4.** Morphological comparison of *Periconiakunmingensis* with other related species. A novel species is indicated by black bold.

Species	Conidiophores	Conidiogenous cells	Conidia	Host occurrence	Origin	Reference
*Periconiacookei*	360–800 µm high, singly or in groups (up to six), 2–6-septate, swollen at the apex, dark brown at the lower part, pale brown at the upper part	7–11 µm diam., spherical, ovoid or pyriform, initially hyaline, smooth-walled, becoming brown, verrucose on age	13–16 µm diam., with the wall up to 2 µm thick, spherical, brown, verrucose, singly or in short chains of 2–3 on conidiogenous cells	On stems of *Heracleumsphondylium*	Great Britain	Mason and Ellis (1953)
(IMI 16174, holotype)						
*Periconiadelonicis*	360−420 μm high, singly, septate, greyish-brown to dark brown, unbranched, smooth to minutely verruculose	Monoblastic, proliferating, ovoid to globose, hyaline	5.5−7 μm diam., subglobose to globose, subhyaline to pale brown, verruculose, singly or in short chains	On pods of *Delonixregia*	Thailand	Jayasiri et al. (2019)
(MFLU 18−2100, holotype)						
*Periconiaelaeidis*	200−400 μm high, singly, 4−7-septate, grayish-brown to dark brown, unbranched, smooth to minutely verruculose	Polyblastic, proliferating, ovoid to globose, pale brown, smooth	4.5−6.5 μm diam., subglobose to globose, subhyaline to pale brown, verruculose, solitary	On dead leaves of oil palm	Thailand	Hyde et al. (2018)
(MFLU 18−0626, holotype)						
***Periconiakunmingensis* (KUN-HKAS 102239, holotype)**	**100–260 μm high, solitary, 3–5-septate, dark brown, unbranched below, branched only at the apex, sometimes swollen near the base**	**(4–)5–8(–10) × 2.5–5(–6) μm, mono- to polyblastic, subspherical to fusiform, subhyaline to pale brown, verruculose**	**4.5–7(–9) × 4–7(–8) μm, subglobose to globose, subhyaline to pale brown, minutely verruculose to short-spinulose, solitary to catenate, in acropetal short chains**	**On dead standing rachis of a fern**	**Yunnan, China**	**This study**
*Periconiapalmicola* (MFLU 14-0198, holotype)	151–188 μm high, singly or in groups, septate, dark brown to black, branched at the apex	3–3.5 × 3–4.8 μm, mono- to polyblastic, globose, hyaline to subhyaline	5.1–7.4 × 4.8–6.1 μm, subglobose to globose, light brown to brown, verruculose, solitary to catenate, in acropetal short chains	On dead, fallen leaves of unidentified palm	Thailand	Hyde et al. (2020)
*Periconiaverrucosa* (MFLU 17–1516, holotype)	170–296 µm high, singly, 2–4-septate, dark brown, with 3–4 short branches at the apex	11–26 × 6–14 μm, mono- to polyblastic, retrogressive, oblong, pale brown	7–15 μm diam., globose, dark brown to reddish-brown, verrucose, acrogenous in branched chains	On dead stems of *Clematisviticella*	Belgium	Phukhamsakda et al. (2020)

## ﻿Discussion

This paper, in the series “Exploring ascomycete diversity in Yunnan”, presents three novel taxa in the suborder Massarineae (Pleosporales), viz. *Bambusicolahongheensis* (Bambusicolaceae), *Periconiakunmingensis* (Periconiaceae) and *Trichobotryssinensis* (Dictyosporiaceae). The novelties of these taxa were well-justified, based on morphological characteristics and phylogenetic evidence, as well as the differences in nucleotide pairwise comparison of reliable genes amongst closely-related taxa. This provides a better fundamental knowledge of the taxonomic framework of ascomycetes in this region.

*Bambusicolahongheensis* is justified, based on multigene phylogeny and the differences in nucleotide pairwise comparison of the ITS region with closely-related species. Monkai et al. (2021) mentioned that many *Bambusicola* species have similar morphology, but these species can be distinguished, based on multigene phylogeny and they also recommended the use of the *rpb2* gene for delineating species level of *Bambusicola*. Unfortunately, the *rpb2* sequence did not distinguish *B.hongheensis* from *B.loculata* in the present study; however, the ITS region of *B.hongheensis* showed > 1.5% nucleotide differences amongst the closely-related species viz. *B.loculata*, *B.massarinia* and *B.triseptatispora*. This provides adequate justification for the species’ novelty following the recommendation of Jeewon and Hyde (2016).

Although many *Bambusicola* species are morphologically somewhat similar, it is notable that they can also be distinguished by their represented asexual morphs that are easily sporulated *in vitro* as well as on natural substrates. For instance, coelomycetous asexual morphs of *B.massarinia* and *B.triseptatispora* sporulated *in vitro*; of which *B.massarinia* can be distinguished from *B.triseptatispora* in having pale brown, 1-septate, cylindrical conidia (Dai et al. 2012). Whereas conidia of *B.triseptatispora* are light brown, 3-septate, cylindrical to cylindrical-clavate (Dai et al. 2017). Unfortunately, the asexual morphs of *B.hongheensis* and *B.loculata* have not yet been determined. Hence, further studies on their asexual morphs sporulated *in vitro* should be carried out for a better understanding through their sexual-asexual reproduction, as well as gaining criteria of species delineation.

*Trichobotryssinensis* is morphologically typical of *Trichobotrys*. *Trichobotrys* was previously classified into Ascomycota genus *incertae sedis* (Wijayawardene et al. 2022b). Although the sequence data of the type species of *Trichobotrys* is currently unavailable, the inclusion of available sequence data along with our new species that morphologically align well with *Trichobotrys* in the phylogenetic analyses, provides compelling evidence supporting the placement of *Trichobotrys* within the Dictyosporiaceae. This information contributes to our understanding of taxonomic relationships and highlights the need for further studies to explore the molecular characteristics and genetic diversity of *Trichobotrys* species within the Dictyosporiaceae.

Synanamorph is the term of use for fungal taxa producing two or more different asexual morphs which were often linked by the sporulation in culture (Wijayawardene et al. 2021a, 2022c). Many fungal taxa have been reported for their synanamorphism, such as *Botryosphaeria* with dichomera-like *in vitro* and *Neofusicoccum* (as *Fusicoccum*) (Barber et al. 2005), *Barbatosphaeriafagi* (≡ *Calosphaeriafagi*) with ramichloridium-like and sporothrix-like asexual morphs (Réblová et al. 2015) and *Synnemasporella* with sporodochial and pycnidial asexual morphs on natural hosts (Fan et al. 2018). The formation of two or more different morphs in a single species has led to misidentification and the distinct morphs have been somehow counted as different species (Wijayawardene et al. 2021a, 2022c). It has further caused problems in the dual nomenclature of pleomorphic fungi that proposed one name for one fungus (McNeill and Turland 2012; Rossman et al. 2015). Interestingly, *Trichobotryssinensis* formed two different asexual morphs, one in nature (as *Trichobotrys*) and another *in vitro* (pycnidial coelomycetous asexual morph) which is the first report of the synanamorphism for the genus *Trichobotrys*. This new finding provides insight into pleomorphism which is essential in further revision of taxonomic boundaries and easing of existing complications. It is noteworthy that *Trichobotrys* formed a well-resolved clade with *Gregarithecium* in the present phylogenetic analyses. Unfortunately, the sexual morph of *Trichobotrys* has not yet been determined. Similarly, the asexual morph of *Gregarithecium* has also not yet been reported. Hence, the sexual-asexual connection between *Gregarithecium* and *Trichobotrys* is doubtful pending future study.

*Periconiakunmingensis* is introduced in this paper, based on its morphology and phylogeny. Morphologically, *P.kunmingensis* fits well with the generic concept of *Periconia* and its phylogenetic affinity is also well-clarified within Periconiaceae. It is noteworthy that the ITS region could not be used to separate *P.kunmingensis* from other closely-related species, including *P.cookei* and *P.verrucosa*, based on the nucleotide pairwise comparison. Whereas, the ITS sequences of *P.delonicis*, *P.elaeidis* and *P.palmicola* are unavailable. The interspecific variation amongst these species may be questionable. However, the *rpb2*, and *tef1-α* gene regions which have sufficient genetic variation can be used to distinguish these species. Nevertheless, the *rpb2* gene of most *Periconia* species is unavailable. Therefore, the sequences of protein-coding genes (e.g. *rpb2* and *tef1-α*) are acquired to offer reliable phylogenetic markers for species delineation.

Over the past five years, the number of newly-described fungal species has been rapidly increasing in Yunnan. Several novel and interesting ascomycetes were described and illustrated from various host plants and on different substrates and habitats. Many studies of ascomycetous taxonomy on specific host substrates have become essential and challenging for mycologists across the region. For instance, D.N. Wanasinghe and his colleagues (2018–2022) carried out research studies on fungal biogeography and published over 40 novel taxa of wood-inhabiting fungi, as well as other substrates in this region (Bao et al. 2019; Wanasinghe et al. 2020, 2021; Yasanthika et al. 2020; Mortimer et al. 2021; Ren et al. 2021; Wijayawardene et al. 2022a; Maharachchikumbura et al. 2022). Simultaneously, S. Tibpromma and her colleagues (2018–2022) have also carried out research studies of fungal taxonomy and diversity on various host plants, such as agarwood, coffee, *Pandanus*, para rubber and tea plants. They introduced 20 novel taxa from *Pandanus* (Tibpromma et al. 2018), while taxonomic studies on the other plants (approximately 45 novel species on agarwood, coffee and para rubber) are pending (S. Tibpromma, personal data information). A comprehensive study of freshwater Sordariomycetes in Yunnan has been carried out by Luo et al. (2018a, b, 2019) who introduced more than 50 novel taxa and reported more than 75 freshwater Sordariomycetes species in Yunnan. Even though these studies unravelled a substantial number of ascomycetes in Yunnan, there is still a huge gap of knowledge in hitherto undescribed novel taxa in this region. If considering only the plant and fungal ratio, many of the so far fungal taxonomic studies on land plants have underestimated these in Yunnan, especially on those economic and horticulture plants. Hence, the inventory of ascomycetes on these land plants will be interesting in further research studies.

## ﻿Conclusion

In conclusion, this study introduces three novel species in the suborder Massarineae (Pleosporales): *Bambusicolahongheensis*, *Periconiakunmingensis* and *Trichobotryssinensis*. These species were found as saprobes in different habitats, with *B.hongheensis* and *P.kunmingensis* occurring in terrestrial environments, while *T.sinensis* was discovered in a freshwater stream. Notably, the presence of *Trichobotrys* in a freshwater habitat is a significant finding, as it aligns with other aquatic lignicolous species within the family Dictyosporiaceae. The novelty of *B.hongheensis* is supported by multigene phylogeny and nucleotide pairwise comparison, although further genetic analysis is recommended. Differentiation between *Bambusicola* species can also be achieved through the examination of their asexual morphs. *Trichobotryssinensis*, morphologically typical of *Trichobotrys*, is phylogenetically placed within Dictyosporiaceae and highlights the need for additional studies on molecular characteristics and genetic diversity within the genus. The observation of synanamorphism in *T.sinensis* adds complexity to its morphological identification and taxonomic boundaries. The introduction of *Periconiakunmingensis* is supported by its morphology and phylogenetic affinity within the family Periconiaceae, although the use of protein-coding genes is recommended for reliable species delineation. This study contributes to our understanding of ascomycete diversity in Yunnan and emphasises the importance of such investigations to enhance our knowledge of newly-discovered taxa.

## Supplementary Material

XML Treatment for Bambusicolaceae

XML Treatment for Bambusicola

XML Treatment for Bambusicola hongheensis

XML Treatment for Dictyosporiaceae

XML Treatment for Trichobotrys

XML Treatment for Trichobotrys sinensis

XML Treatment for Periconiaceae

XML Treatment for Periconia

XML Treatment for Periconia kunmingensis

## ﻿Additional information

### Author ORCIDs

Rungtiwa Phookamsak https://orcid.org/0000-0002-6321-8416

Sinang Hongsanan https://orcid.org/0000-0003-0550-3152

Darbhe Jayarama Bhat http://orcid.org/0000-0002-3800-5910

Dhanushka N. Wanasinghe https://orcid.org/0000-0003-1759-3933

Itthayakorn Promputtha https://orcid.org/0000-0003-3376-4376

Nakarin Suwannarach https://orcid.org/0000-0002-2653-1913

Jaturong Kumla https://orcid.org/0000-0002-3673-6541

Ning Xie https://orcid.org/0000-0002-5866-8535

Turki M. Dawoud http://orchid.org/0000-0002-1444-4185

Peter E. Mortimer https://orcid.org/0000-0002-8507-7407

Jianchu Xu https://orcid.org/0000-0002-2485-2254

Saisamorn Lumyong https://orcid.org/0000-0002-6485-414X

### Data availability

All of the data that support the findings of this study are available in the main text.
